# Assessing the potential of native ecotypes of *Poa pratensis* L. for forage yield and phytochemical compositions under water deficit conditions

**DOI:** 10.1038/s41598-022-05024-1

**Published:** 2022-01-21

**Authors:** Nikwan Shariatipour, Bahram Heidari, Zahra Shams, Christopher Richards

**Affiliations:** 1 Department of Plant Production and Genetics, School of Agriculture, Shiraz University, 7144165186 Shiraz, Iran; 2 Department of Horticulture Science, School of Agriculture, Shiraz University, Shiraz, Iran; 3 USDA ARS National Laboratory for Genetic Resources Preservation, Fort Collins, CO USA

**Keywords:** Plant breeding, Plant stress responses, Secondary metabolism

## Abstract

Evaluation of forage yield and antioxidant activity in *Poa pratensis* with high quality and good spring green-up forage might help variety improvement for use under water deficit condition. Germplasm and phenotypic diversity evaluations lay a foundation for genotype selection and improvement of varieties for drought tolerance in *P. pratensis*. The present study was conducted to assess the genetic potential of a collection of *P. pratensis* accessions for drought stress and to identify the association between polyphenol compounds and forage yield traits. Vegetative clone samples of 100 accessions collected from a diverse geographical area of Iran were clonally propagated in a greenhouse and evaluated in the field under two moisture regimes (non-stress and drought stress) in 2018 and 2019. Drought stress had negative effects on fresh and dry forage yields and reduced phenotypic variances. On average, drought stress reduced fresh and dry forage yields by 45% and 28%, respectively. The results of Mantel test showed no significant correlation between forage yield traits and geographical distances. Genetic coefficients of variation for forage yield and most of the phytochemicals were lower under drought stress, suggesting that deficit irrigation may reduce genetic variation for the tested traits. The estimates of heritability were higher under non-stress conditions than under drought stress treatment for forage yield traits and few polyphenols. However, the majority of polyphenol compounds had higher heritability than forage yield traits under drought stress, which suggests the potential for indirect selection. The ‘Ciakhor’, ‘Damavand’, ‘Karvandan’, ‘Abrumand’, and ‘Abr2’ accessions had high quantities for polyphenols and yield traits under both moisture regimes. These accessions are promising candidates for use in variety crossing programs and for developing high-yielding varieties under water-deficit conditions.

## Introduction

The sessile nature exposes plants to changing environments^1,2^. Drought and heat stresses are the environmental side effects of climate change caused by increasing greenhouse gases^3^. Drought stress has severe and direct effects on plant growth and productivity^4–7^. Drought stress induces oxidative stress, which leads to changes in cellular redox homeostasis and excessive generation of reactive oxygen species (ROS)^8,9^. Accumulation of ROS results in the destabilization and peroxidation of plant cell components^9,10^. However, plants have evolved endogenous defensive mechanisms to deal with oxidative stress through non-enzymatic antioxidants consisting of polyphenolic compounds^11–14^. The accumulation of polyphenolic compounds and enhanced antioxidant activities increase plant tolerance to abiotic stress^15^. Furthermore, the presence of polyphenol compounds in diets has positive effects on the productive performance and health of livestock^16–18^. Phytochemically rich forage sources, especially polyphenolic compounds, have health benefits for livestock, humans, and the environment^18,19^.

Over 82% of the Iran agricultural regions is mainly arid and semi-arid with high temperatures exceeded 40 °C except the northern coastal areas^20^. The climate is extremely continental with hot and dry summer and very cold winter particularly in inland areas. Apart from the coastal areas, the temperature in Iran is characterized by relatively large annual range varying between 22 and 26 °C. The average annual rainfall of the country is about 240 mm^21^ which shows water scarcity specifically at the reproductive stages of crop plants. In some years, Iran experienced higher-than-average precipitation, but long-term drought conditions persisted^22,23^. Long-term historical climate records revealed that some Iran provinces have been affected by moderate, severe, and extreme droughts. Despite these conditons and due its habitat diversity, Iran continues to maintain high species diversity with over 8000 recorded^24^. This ecogeographic diversity may impart strong ecotypic differentiation in widespread species like *P. pratensis* (Kentucky bluegrass). The *P. pratensis* L. with high quality and good spring green-up forage is an important forage crop belonging to the Poaceae family that includes more than 500 described species viz. forage cereals and turf grasses^25–27^. The morphological, cytological, and species diversity of Kentucky bluegrass show that it originated mainly from Europe and Asia^28–30^. The genetic diversity among *P. pratensis* ecotypes might assist in breeding for drought tolerance. The abundance of potential *P. pratensis* ecotypes in Iran may offer genetic critical resilience to climate change, which is a challenge for agriculture in the region. Germplasm evaluation including assessment of available phenotypic diversity and estimation of the heritability of desired traits is a preliminary step in the early phase of breeding programs to provide raw material from genetic resources and develop high forage yield varieties with higher antioxidant activity and polyphenol components^31,32^. The evaluation of Kentucky bluegrass germplasm through the analysis of molecular markers has been previously documented^33–36^. Previous studies show that physiological traits, the plant persistence and recovery and antioxidant enzyme activities have been assessed in lawn Kentucky bluegrass varieties under drought stress^37–39^. However, these studies focused on the lawn perspective of this species and the assessment of variation in forage yield traits and phytochemical compositions is missing in such studies. Forage is the most important source of fibrous energy and maybe directly consumed via grazing. A very important property of forage grasses is the ability to give stable and high dry matter yield under different environmental conditions. Analysis of genetic diversity is prerequisite for selection and identify drought adaptive traits in forage grasses that helps to improve drought tolerance and identify valuable genes for marker-assisted selection in grasses. Therefore, the aim of the current study was to assess the genetic diversity of a collection of *P. pratensis* accessions for forage yield and phytochemical compositions under drought stress conditions and to pre-breed possible drought tolerant candidates for use in breeding programs.

## Material and methods

### Plant material and field experiments

**Figure 1:**
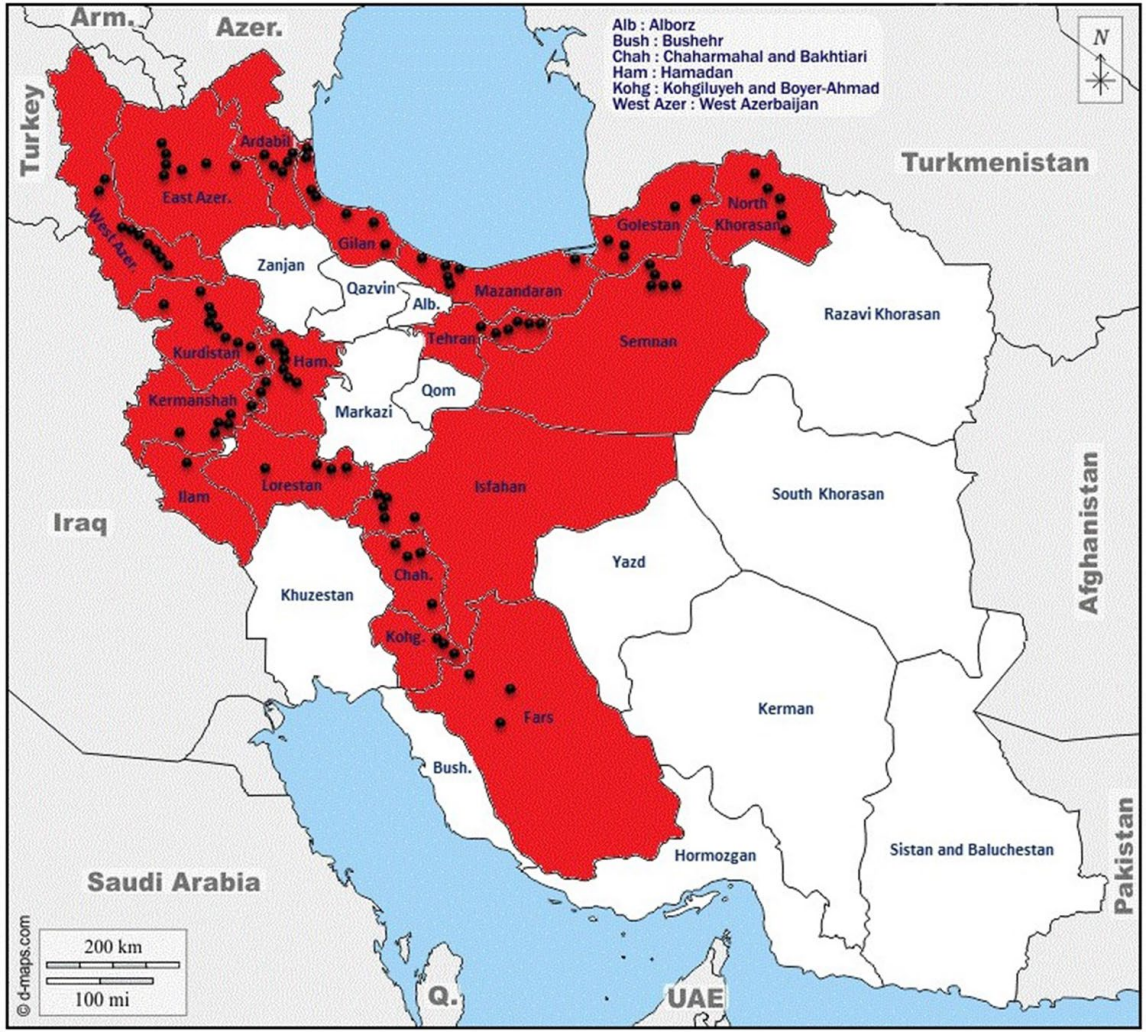
The collection areas of *Poa pratensis* accessions in Iran. The red color indicated provinces and the block circles represent the approximate location of the collected accessions. The original map obtained from d-map (https://d-maps.com/carte.php?num_car=5496&lang=en) and modified (colored) using Adobe Photoshop CS6.

The map of locations where 176 Kentucky bluegrass ecotypes were collected by the first and second authors from the wild in a wide geographical area in Iran is shown in Fig. 1. The plant samples that are not considered as threatened species and not listed as species with small or very small populations in Iran, were identified in the Laboratory of Department of Plant Production and Genetics following the NCBI Taxonomy description (https://www.ncbi.nlm.nih.gov/Taxonomy/Browser/wwwtax.cgi?lvl=0&id=4545). The source of plant materials with the voucher ID of NS-BH-POP1400 was deposited in the Seed Bank of Department of Plant Production and Genetics, School of Agriculture, Shiraz University, Iran and are available for research purposes. All experiments including the collection and use of plant samples were conducted according to the relevant institutional, national, and international guidelines and legislation. The clone samples collected from a depth of 40 cm of soil and plants containing 10 to 15 tillers transferred to plastic pots. First, the plant samples were clonally propagated in a greenhouse at the School of Agriculture, Shiraz University, Iran. After pre-evaluation of the samples, 100 viable and established accessions were used for further evaluation under field conditions.

This experiment was carried out in the field at the Research Farm of Shiraz University, located in Bajgah, Shiraz, Fars, Iran (52° 35 N and 39° 4 E, 1810 amsl) in the 2017–2018 and 2018–2019 seasons. The long-term mean of maximum (22.95 °C) and minimum (4.9 °C) temperatures and mean annual precipitation of 394 mm generally without rain during the summer made supplemental irrigation necessary for growing crops during this period. The characteristics and geographic information of the collection areas are presented in Fig. 1 and Supplementary Table S1. The germplasm panel used for field evaluations consisted of 100 clonally propagated plants transferred to the field in March 2017 and were grown in two separate field experiments. The total depth of the soil profile was classified as clay loam (fine, mixed, mesic, Typic Calcixerepts). After field establishment, accessions were evaluated under well-watered (non-stress) and water deficit irrigation (drought stress) in a randomized complete block design (RCBD) with two replicates. Each experimental plot consisted one clone cultivated on 1 m^2^ space with a 80 cm between plot distance. The physical characteristics of the soil were measured for the implementation of the drought stress treatment (Table 1). Full irrigation (100% gross irrigation water, *dg*) and 50% *dg* were considered as non-stress and drought stress treatments, respectively. First, the net irrigation depth ($${d}_{n})$$ was measured using the soil water content in the root zone before irrigation ($${\theta }_{i})$$ as follows (Eq. 1)^40^:

**Table 1:** The physical characteristic of soil in the field used for evaluation of genetic diversity in *Poa pratensis* accessions.

Parameter	Unit	Soil depth (cm)	
		0–30	30–60
Field capacity (FC) (− 0.033 MPa)	cm^3^ cm^−3^	32	33
Permanent wilting point (PWP) (− 1.5 MPa)	cm^3^ cm^−3^	11	16
Bulk density (BD)	g cm^−3^	1.31	1.37
Clay	%	36	39
Sand	%	25	27
Silt	%	39	34
Texture	–	Clay loam	Clay loam

1 $${d}_{n}=\sum_{i=1}^{n}\left({\theta }_{FCi}-{\theta }_{i}\right)\times {\Delta z}_{i}$$

where ${d}_{n}$ is the net irrigation water depth (m), n is the number of soil layers, ${\theta }_{FCi}$ is the volumetric soil water content in layer i at field capacity, ${\theta }_{i}$ is the volumetric soil water content in layer *i* before irrigation, and ${\Delta z}_{i}$ is the thickness of soil in layer *i* (m). Then, an irrigation application efficiency of 90% (most commonly used in drip irrigation) used to determine the gross irrigation water (${d}_{g}$) (Eq. 2)^40^:

2 $${d}_{g}={d}_{n}/0.9$$

where, for the non-stress treatment, 100% $${d}_{g}$$ and for the drought stress 50% $${d}_{g}$$ were applied^40^. In drought stress treatment, 50% gross irrigation water ($${d}_{g})$$ provided through crop growth cycle exerted water deficit conditions.

### Forage traits assay

Forage yield was determined by measuring forage fresh yield (FFY) and forage dry yield (FDY) in both years. The FFY measured as the weight of fresh herbage harvested per plot and FDY as the dried herbage after drying at 72˚C for 48 h.

### Phytochemical assay

#### Methanolic extract preparation

For the extract preparation, the plant materials were ground in a mechanical grinder after drying at room temperature. Then, 20 g of each sample was mixed with 200 mL of methanol and macerated for 24 h in a shaker at room temperature. The extract was centrifuged at 10,000 g for 10 min at 4 °C, and the supernatant was filtered with grade 1 Whatman paper (Whatman International Ltd., Maidstone, England). The resulting extracts were stored for further assays.

#### Antioxidant activity

Antioxidant activity was evaluated using the 1, 1-dipheny l-2-picrylhydrazyl (DPPH) free radical scavenging activity according to the method described by Ao et al.^41^. Briefly, 50 μL of the methanolic extract was added to 950 μL DPPH that vortexed for 30 s and kept at room temperature in the dark for 15 min. The absorbance of the samples was measured at 515 nm using a spectrophotometer (Epoch microplate spectrophotometer, USA). The antioxidant activity expressed as the percentage of decline in absorbance, compared to that of the control, and corresponded to the percentage of scavenged DPPH. The percentage of scavenged (% DPPHsc) was calculated using Eq. (3):

3 $$\mathrm{\% }{\mathrm{DPPH}}_{\mathrm{sc}}=\frac{\left(Ac-\mathrm{As}\right)\times 100}{\mathrm{Ac}}$$

where, ‘A_c_’ and ‘A_s_’ are the absorbances of the control and the sample, respectively.

#### Total phenol content (TPC)

The TPC measured following a modification of the Folin-Ciocalteu colorimetric method^42,43^. Briefly, 1 mL of the extract mixed with 1 mL hydrochloric acid (6 mol) and 5 mL of methanol (75%). Then, 1 mL of the solution was reacted with 5 mL of 10% Folin-Ciocalteu reagent and alcalinized with Na_2_CO_3_. The absorbance of the mixture measured at 760 nm after 90 min using a spectrophotometer (Epoch microplate spectrophotometer, USA). Gallic acid was used as a standard (Eq. 4), and the TP results were expressed as mg gallic acid/100 g of dry weight:

4 $$Y=0.01X-0.0101, {R}^{2}=0.9934$$

where Y and X denote the total phenol content and absorbance at 760 nm, respectively.

#### Total flavonoid content (TFC) and flavanone

The TFC was determined based on a modified method for aluminum chloride complex^44^. Briefly, the 0.5 mL methanolic extracts of each sample were combined with 1.5 ml methanol, 0.1 ml aluminum chloride (% 10 methanol), 0.1 mL potassium acetate (1 M) and 2.8 mL distilled water, separately. The solutions were then incubated at room temperature for 30 min. Then, the absorbance of each reaction mixture was measured at 510 nm using a spectrophotometer (Epoch microplate spectrophotometer, USA). Finally, the catechin standard curve was used to determine catechin equivalents (CE, mg) per 100 g dry weight. Flavanone was measured using Popova et al., method^45^, and the absorbance readings of each reaction mixture at 486 nm was obtained by a spectrophotometer (Epoch Microplate Spectrophotometer, BioTek Instruments, Inc., USA) and expressed as mg/100 g dry weight.

#### Total anthocyanin content

The dried samples were mixed with 10 mL of methanol–water-concentrated HCl (80:20:1). The samples placed on a shaker in a dark room at 4 °C for 48 h. After 48 h, the extracts filtered with grade 1 Whatman paper (Whatman International Ltd., Maidstone, England). The absorbance reads were obtained using a spectrophotometer (Epoch Microplate Spectrophotometer, BioTek Instruments, Inc., USA) at wavelengths of 530 and 657 nm. Finally, the anthocyanin content was measured using Eq. (5) and expressed as mg/100 g dry weight^46,47^.

5 $$A = {A}_{530}-0.33 {A}_{657}$$

### Statistical analyses

Analysis of variance was carried out to test the years, water regimes, accessions, and their interactions. The variance components were estimated using a general linear model (GLM) (Table 2). The effect of year was defined as random, whereas accession and irrigation regime were considered as fixed in the GLM model.

**Table 2:** Expected mean squares for phytochemicals and forage yield traits across two environments (non-stress and drought stress) and in 2018 and 2019 seasons for evaluation of *Poa pratensis* accessions.

Source of variation	Degree of freedom	Expected mean squares
Block	r − 1 = 1	
Genotype	g − 1 = 99	${\sigma }_{e}^{2}+{r\sigma }_{g}^{2}$
Error	(r − 1) (g − 1) = 99	${\sigma }_{e}^{2}$

g, genotype; r, number of block, ${\sigma }_{e}^{2}$, error variance; ${\sigma }_{g}^{2}$, genotypic variance.

Broad-sense heritability (${\mathrm{h}}^{2}$), phenotypic coefficients of variation (PCV), and genotypic coefficients of variation (GCV) were estimated according to the following equation (Eqs. 6–12)^48^:

6 $${\upsigma }_{\mathrm{g}}^{2}=\frac{{MS}_{g}-{MS}_{e}}{r}$$

7 $${\upsigma }_{\mathrm{e}}^{2}={MS}_{e}$$

8 $${\upsigma }_{P}^{2}={\sigma }_{g}^{2}+{\sigma }_{e}^{2}$$

9 $${\mathrm{h}}^{2}=\frac{{\sigma }_{g}^{2}}{{\sigma }_{p}^{2}}$$

10 $$\mathrm{GCV}=\frac{\sqrt{{\sigma }_{g}^{2}}}{\mu }\times 100$$

11 $$\mathrm{PCV}=\frac{\sqrt{{\sigma }_{p}^{2}}}{\mu }\times 100$$

12 $${\mathrm{SE}}_{{\mathrm{h}}^{2}}=\sqrt{\frac{2{{(MS}_{e})}^{2}(\frac{1}{dfe+2}+\frac{1}{dfg+1})}{({MSg)}^{2}}}$$

where *g*, ${\sigma }_{g}^{2}$, ${\sigma }_{p}^{2}$, ${\sigma }_{e}^{2}$, ${\mathrm{SE}}_{{\mathrm{H}}^{2}}$, ${\mathrm{MS}}_{\mathrm{e}}$, $\mathrm{MSg}$, dfe, dfg, and $\mu$ are the number of accessions, genotypic variance, phenotypic variance, error variance, standard error of heritability, mean square of the error, mean square of the genotype, error degree of freedom, genotype degree of freedom, and mean of the traits tested, respectively.

The combined data of forage yield and phytochemical traits over the two years were used for multivariate analyses. Simple correlation coefficients were estimated to determine the associations between traits. The genotypic correlations were estimated from the variance components obtained based on the expected mean squares (Eq. 13):

13 $${\mathrm{r}}_{\mathrm{g}(\mathrm{XY})}=\frac{{S}_{g(XY)}}{{S}_{g(X)}\times {S}_{g(Y)}}$$

where ${\mathrm{r}}_{\mathrm{g}(\mathrm{XY})}$ is the genotypic correlation between traits X and Y, ${S}_{g(XY)}$ is the genotypic covariance between traits X and Y, ${S}_{g(X)}$ is the genotypic variance of trait X, and ${S}_{g(Y)}$ is the genotypic variance of trait Y. Stepwise regression was used to determine the most important variables accounting for the forage yield variability^49^. A heatmap was constructed, followed by cluster analysis using a shiny heat map tool based on Manhattan distance metrics and ward.D2 linkage algorithm, respectively [^50^; http://shinyheatmap.com/]. The data used for heatmap construction were normalized based on Z-scores as follows (Eq. 14),

14 $$Z=\frac{X-\mu }{\sigma }$$

where, $X$, $\mu$ and $\sigma$ are the raw data, mean, and standard deviation, respectively, for each trait tested. To test the correlation between geographical data and forage yield of the studied accessions, we conducted a Mantel test in R using 10,000 permutations. The Mantel test for analysis of the association between yield traits and geographical information matrices was performed as follows^51^ (Eq. 15):

15 $${Z}_{m}=\sum_{i=1}^{n}\sum_{j=1}^{n}{X}_{ij}\times {Y}_{ij}$$

where ${X}_{ij}$ and ${Y}_{ij}$ are the forage yield and geographic distance between individuals *i* and *j*, respectively.

Statistical analyses were performed using SAS version 9.4 (SAS Institute, Cary, NC, USA), Minitab version 18 (Minitab, LLC), IBM SPSS version 24 (IBM Corp, Armonk, NY) and R (*vegan*^52^, *geosphere*^53^) packages were used.

## Results

### Effect of drought on traits

**Figure 2:**
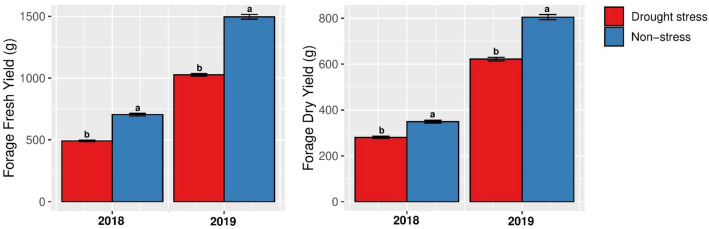
Mean for fresh and dry forage yields of 100 *Poa pratensis* accessions evaluated in non-stress and drought stress conditions in 2018 and 2019. Different letters (*a, b*) represent significant differences between non-stress and drought stress condition in each year at *P* < 0.001.

The results of the analysis of variance showed that the effects of year (Y), irrigation regime (M), genotype (G), irrigation regime × genotype (M × G), year × genotype (Y × G) and year × genotype × irrigation regime (Y × G × M) were significant for all traits (Table 3). In addition, the effect of year × irrigation regime (Y × M) was also significant for all of the assessed traits expect for Antho (Table 3). Mean comparisons of fresh and dry forage yields for the two environments over the years are presented in Fig. 2.

**Table 3:** Mean squares and source of variations in combined analysis of variance for traits evaluated in non-stress and drought stress in 100 of *Poa* *pratensis* accessions in 2018 and 2019.

Characters	Irrigation regime (M) (df = 1)	Error-I (df = 2)	Genotype (G) (df = 99)	(M × G) (df = 99)	Error-II (df = 198)	Year (Y) (df = 1)	Y × M (df = 1)	Y × G (df = 99)	Y × G × M (df = 99)	Residual (df = 200)
TPC	36,800.80***	4.96^n.s^	3256.28***	88.52***	4.84***	91,833.74***	2712.51***	70.23***	57.40***	2.40
TFC	135,225.14***	51.53^n.s^	40,896.10***	593.80***	36.21***	239,114.72***	654.65***	330.35***	285.11***	19.62
FLv	17,445.57***	0.86^n.s^	3468.88***	115.83***	1.34^n.s^	40,725.78***	151.71***	23.78***	32.78***	1.14
Anti	6012.30***	2.16^n.s^	1184.83***	53.29***	1.06^n.s^	19,306.27***	95.79***	20.10***	10.44***	1.49
Antho	4052.25***	3.26^n.s^	555.89***	39.58***	1.47^n.s^	11,437.31***	2.57^n.s^	17.10***	13.29***	1.38
FFY	23,271,035.52***	5115.25^n.s^	116,521.07***	45,513.89***	5482.94***	87,757,778.53***	3,314,064.13***	39,434.88***	40,753.07***	3020.60
FDY	3,162,499.07***	898.29***	50,298.39***	14,875.64***	2200.59*	31,668,496.63***	657,526.96***	15,778.87***	11,865.47***	1711.00

n.s, * and *** represent non-significant and significant at P < 0.05 and P < 0.001, respectively. M, moisture environment; G, genotype; Y, year, error I = R(M); EII = R × G(M); TPC, total phenol content; TFC, total flavonoid content; FLv, flavanone; Anti, antioxidant activity; Antho, anthocyanin; FFY, forage fresh yield; FDY, forage dry weight.

Although drought reduced fresh and dry forage yield in both years, accessions presented higher forage yield in the second year, most likely due to the larger plant size (Fig. 2). Fresh forage yield reduced by 43% and 46% under drought stress in 2018 and 2019, respectively. Dry forage yield also decreased by 24% (2018) and 29% (2019) under drought-stress conditions in 2018 and 2019, respectively (Table 4). However, TPC, TFC, FLv, anthocyanin, and antioxidant activity were increased in response to drought stress (Table 4).

**Table 4:** Mean values, genotypic coefficients of variation (GCV), phenotypic coefficients of variation (PCV) and broad-sense heritability (h^2^) of studied traits measured from one-hundred accessions of *Poa pratensis* evaluated in non-stress and drought stress environments during years 2018 and 2019.

Trait	Mean ± SE (2018)			Non-stress condition					
				GCV (%)		PCV (%)		h^2^ ± SE	
	Non-stress condition	Drought stress	Difference (%)	2018	2019	2018	2019	2018	2019
TPC	65.12 ± 1.18	75.00 ± 1.49	13.18	25.54	27.13	25.75	27.26	0.9833 ± 0.00167	0.9905 ± 0.00095
TFC	201.54 ± 4.05	229.35 ± 5.36	12.13	28.32	31.89	28.48	31.98	0.9887 ± 0.00113	0.9940 ± 0.00060
FLv	63.47 ± 1.40	73.68 ± 1.55	13.86	31.15	26.73	31.18	26.77	0.9978 ± 0.00022	0.9967 ± 0.00033
Anti	52.16 ± 0.81	56.95 ± 0.88	8.41	22.04	20.50	22.10	20.56	0.9948 ± 0.00052	0.9938 ± 0.00062
Antho	34.46 ± 0.55	38.85 ± 0.62	11.29	22.56	20.96	22.77	21.22	0.9815 ± 0.00186	0.9754 ± 0.00248
FFY	704.91 ± 11.08	492.53 ± 6.33	-43.12	19.62	17.54	22.26	18.23	0.7770 ± 0.02497	0.9258 ± 0.00767
FDY	349.11 ± 6.06	280.70 ± 4.76	-24.37	19.97	19.35	24.61	20.19	0.6583 ± 0.04101	0.9188 ± 0.00842

Trait	Mean ± SE (2019)			Drought stress					
				GCV (%)		PCV (%)		h^2^ ± SE	
	Non-stress condition	Drought stress	Difference (%)	2018	2019	2018	2019	2018	2019
TPC	82.86 ± 1.59	100.11 ± 1.59	17.23	27.99	22.48	28.07	22.53	0.9947 ± 0.00053	0.9948 ± 0.00052
TFC	237.92 ± 3.37	262.12 ± 5.58	9.23	33.07	30.12	33.13	30.17	0.9967 ± 0.00033	0.9967 ± 0.00033
FLv	78.61 ± 1.48	87.08 ± 1.59	9.73	29.70	25.86	29.73	25.90	0.9977 ± 0.00023	0.9970 ± 0.00030
Anti	61.30 ± 0.89	67.47 ± 0.97	9.15	21.77	20.32	21.90	20.41	0.9880 ± 0.00120	0.9920 ± 0.00080
Antho	41.91 ± 0.63	46.53 ± 0.69	9.92	22.64	20.96	22.78	21.14	0.9872 ± 0.00128	0.9835 ± 0.00166
FFY	1496.05 ± 19.24	1026.22 ± 10.59	− 45.78	14.25	13.67	18.21	14.62	0.6122 ± 0.04787	0.8733 ± 0.01346
FDY	804.37 ± 11.46	621.29 ± 7.73	− 29.47	19.48	16.43	24.01	17.64	0.6582 ± 0.04103	0.8680 ± 0.01406

TPC, total phenol content; TFC, total flavonoid content; FLv, flavanone; Anti, antioxidant activity; Antho, anthocyanin; FFY, forage fresh yield; FDY, forage dry weight; SE, standard error of the mean.

The highest increase in phytochemicals belonged to FLv, which was 14% under drought stress in the first year, followed by TPC (13%), TFC (12%), and Antho (11%). The antioxidant activity demonstrated a lower increase (8%) in the first year of drought stress treatment. On the other hand, TPC with a 17% increase was higher under drought stress in the second year, followed by Antho and FLv with 10% and TFC and Anti with 9% (Table 4).

Total phenol content (TPC) ranged from 40.12 to 139.81 mg/100 g DW. ‘Damavand’ in second year under drought stress treatment and ‘Gilan-tapeh’ under non-stress environment in the first year had the highest and lowest TPC, respectively (Supplementary Table S2). ‘Ciakhor’ (139.41 mg/100 g DW) and ‘Karvandan’ (139.21 mg/100 g DW) stood at the second and third rankings for TPC under drought stress in 2019. The total flavonoid content (TFC) ranged from 92.53% to 399.5 mg/100 g DW. The ‘Ciakhor’ in second year and under drought stress condition illustrated the highest TFC followed by ‘Karvandan’ (398.10 mg/100 g DW) and ‘Damavand’ (392.44 mg/100 g DW) under drought stress of 2019 experiment (Supplementary Table S2). ‘Karvandan’ and ‘Abbasabad’ showed the highest and lowest flavanone content under drought stress in 2019 and non-stress condition in 2018.

As presented in Supplementary Table S2, the antioxidant activity varied from 29.61 to 89.06% among the accessions studied. ‘Ciakhor’ (89.06 mg/100 g DW) and ‘Karvandan’ (88.85 mg/100 g DW) showed higher antioxidant activity under drought stress in 2019wherease the lowest antioxidant activity was belonged to ‘Karimabad’ (29.61%) (Supplementary Table S2). Total anthocyanin content ranged from 68.29 mg/100 g DW in the ‘Karvandan to 17.03 mg/100 g DW in the ‘Abbasabad’ in 2019 under drought stress condition (Supplementary Table S2). The fresh and dry forage yields ranged from 332.50 g to 2026.57 g (forage fresh yield (FFY)) and 175.24 g to 1129.00 g (forage dry yield (FDY)). ‘Ciakhor’ in 2019 under non-stress condition and ‘Gilan-tapeh’ under drought stress condition in 2018 had the highest and lowest fresh and dry forage yield, respectively (Supplementary Table S2).

### Heritability and genotypic variations in *P. pratensis*

Genotypic and phenotypic coefficients of variation (GCV and PCV) for the non-stress and drought treatments over the years are presented in Table 4. In 2018, the GCV ranged from 19.62% (FFY) to 31.15% (FLv) under irrigation conditions and from 14.25% (FFY) to 33.07% (TFC) under drought treatment. In 2019, TFC (31.89%, 30.12%) and FFY (17.54%, 13.67%) showed the highest and lowest GCV under non-stress and drought treatments, respectively. Similar results showed that FLv and TFC had the highest PCVs in both moisture regimes. FFY had the lowest PCV in both the years (Table 4).

In 2018, the heritability estimates varied from 65.83% (FDY) to 99.78% (FLv) under non-stress conditions and from 61.22% (FFY) to 99.77% (FLv) under drought stress (Table 4). The heritability of the antioxidant activity (h^2^ = 99.48%) under non-stress conditions and TFC (h^2^ = 99.67%) and TPC (h^2^ = 99.47%) under drought stress conditions stood at the next rankings. In 2019, heritability ranged from 91.88% for FDY to 99.67% for FLv under non-stress conditions and from 86. to 99.70% for the same traits under drought stress conditions. The forage traits (FFY and FDY) showed higher heritability in 2019 than in 2018 in the two moisture regimes (Table 4). Furthermore, the phytochemical traits had high heritability with slight change between non-stress and drought conditions over years, whereas heritability of FFY and FDY in 2018 was moderate in both moisture conditions. The FFY and FDY traits represented lower heritability under drought stress than non-stress condition in 2019.

### Relationship of traits

**Figure 3:**
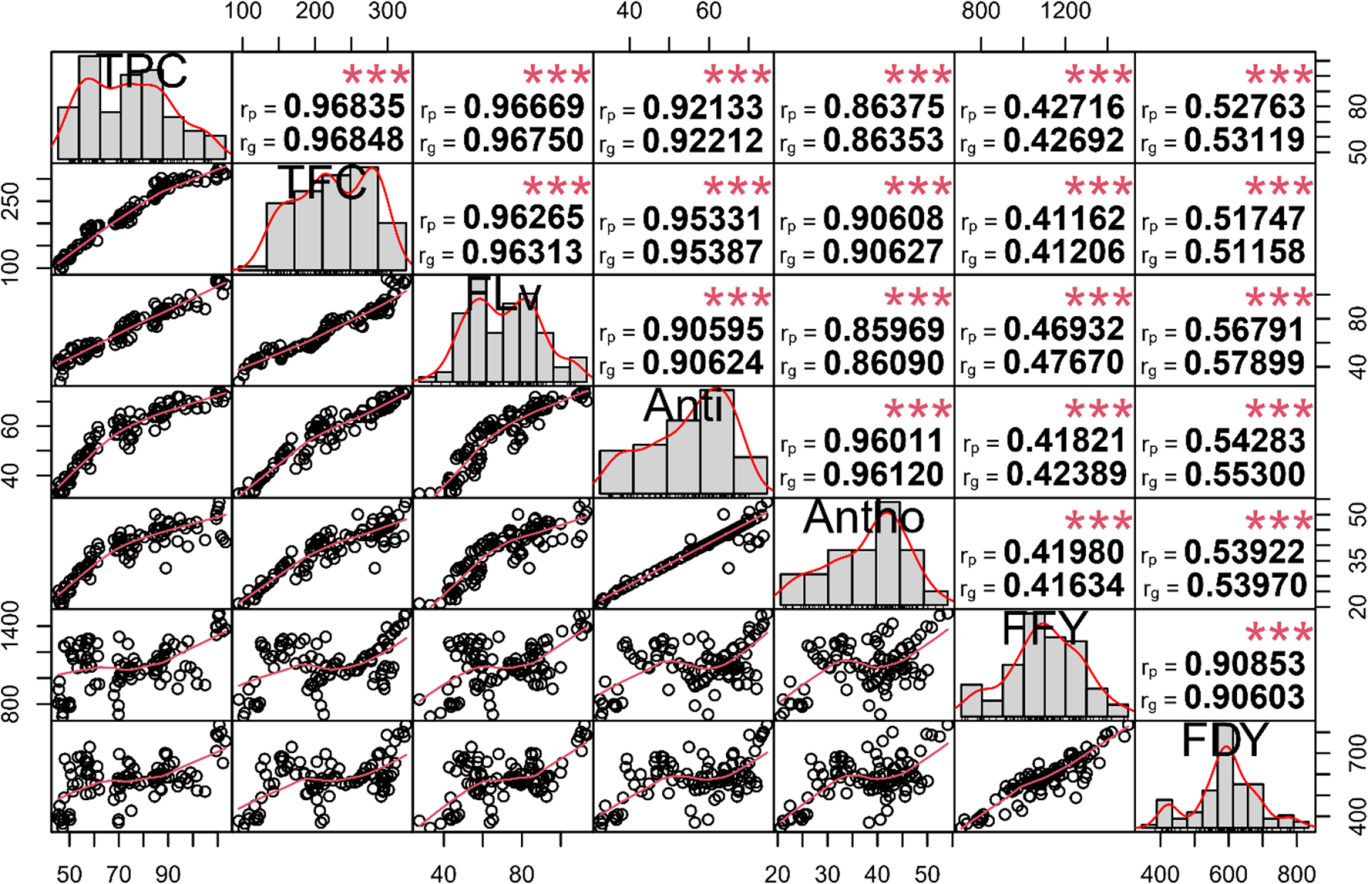
Phenotypic (r_p_) and genotypic (r_g_) correlation coefficients for traits in 100 *Poa pratensis* accessions evaluated in non-stress conditions.

**Figure 4:**
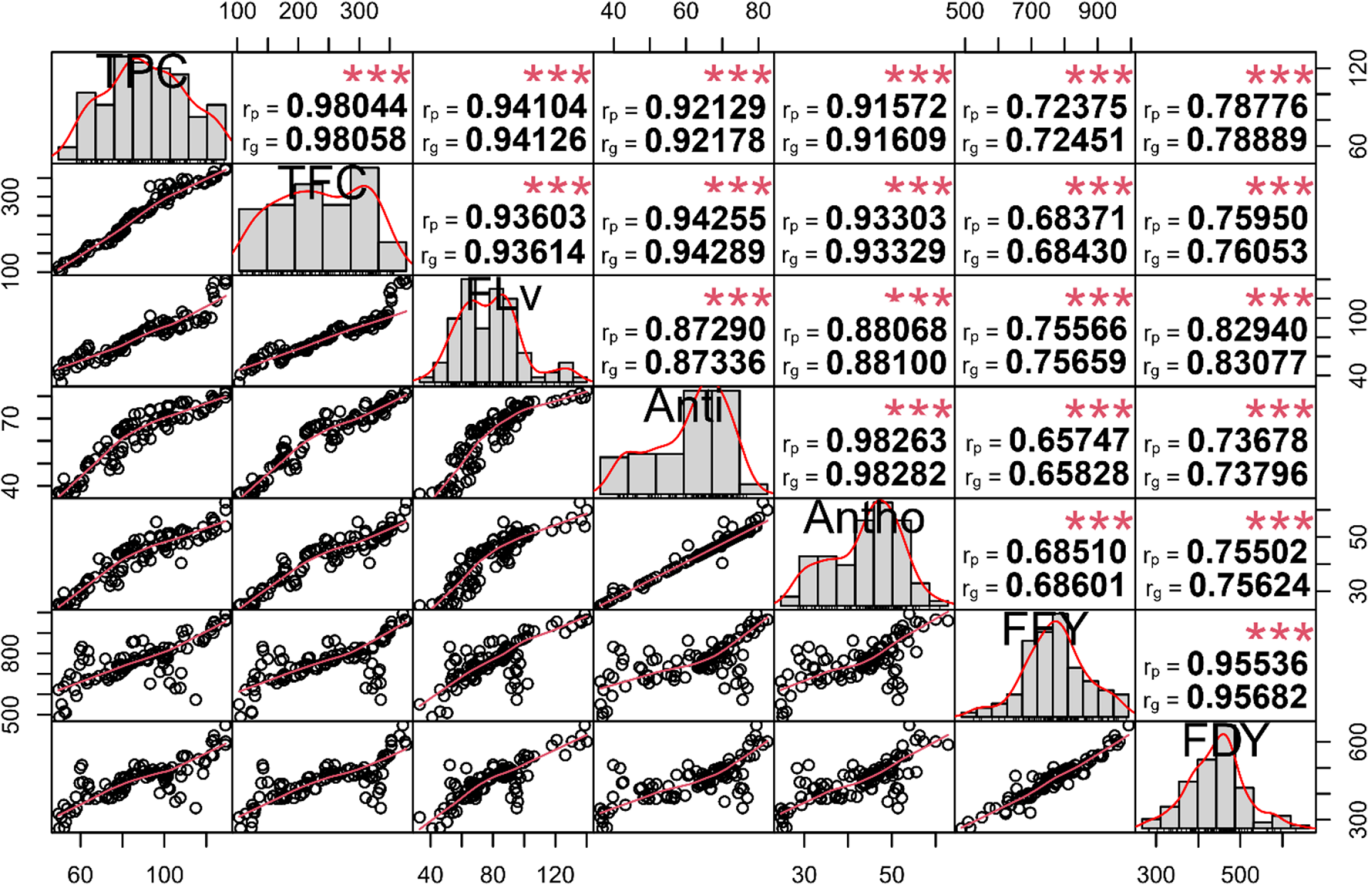
Phenotypic (rp) and genotypic (rg) correlation coefficients for traits in 100 of *Poa pratensis* accessions evaluated in drought stress conditions.

The correlation coefficients of phytochemical and forage yield traits under both non-stress and drought stress conditions are shown in Figs. 3 and 4, respectively. Antioxidant was strongly correlated with Antho (non-stress, r_p_ and r_g_ = 0.96; drought stress, r_p_ and r_g_ = 0.98) and TFC (non-stress, r_p_ and r_g_ = 0.95; drought stress, r_p_ and r_g_ = 0.94). Polyphenols were significantly correlated. For instance, TPC was strongly correlated with TFC (non-stress, r_p_ and r_g_ = 0.97; drought stress, r_p_ and r_g_ = 0.98), FLv (non-stress, r_p_ and r_g_ = 0.97; drought stress, r_p_ and r_g_ = 0.94) and Antho (non-stress, r_p_ and r_g_ = 0.87; drought stress, r_p_ and r_g_ = 0.92). Antho showed high correlation with TFC (non-stress, r_p_ and r_g_ = 0.90 ; drought stress, r_p_ and r_g_ = 0.93) and FLv (non-stress, r_p_ and r_g_ = 0.86; drought stress, r_p_ and r_g_ = 0.88). The results of correlation analysis indicated that TFC and FLv had high genotypic (non-stress, r_g_ = 0.96; drought stress, r_g_ = 0.94) and phenotypic (non-stress, r_g_ = 0.96; drought stress, r_g_ = 0.94) correlations. The Anti and FLv traits did significantly correlate under both non-stress (r_p_ and r_g_ = 0.91) and drought stress (r_p_ and r_g_ = 0.87) conditions.

Additionally, FFY was strongly correlated with FDY under both non-stress (r_p_ and r_g_ = 0.91) and drought stress conditions (r_p_ and r_g_ = 0.96). Correlation of the antioxidant activity and polyphenolic components with fresh and dry forage yield was stronger under drought treatments than under non-stress conditions. The results of stepwise regression showed that flavanone and total flavonoid content were the most important contributors to fresh forage yield (R^2^ = 24%), whereas flavanone content, total flavonoid content, and antioxidant activity explained the highest variation in dry forage yield (R^2^ = 38%) under non-stress conditions (Table 5). Under drought stress treatment, 62% of the variation in fresh forage yield and 72% of the dry forage yield phenotypes were explained by flavanone, total flavonoid, total anthocyanin, and total phenol content (Table 5).

**Table 5:** Results of stepwise regression analysis for predicting fresh and dry forage yield contributors in *Poa pratensis* accessions evaluated in non-stress and drought stress conditions.

Treatment	FFY					FDY				
	Variable entered	Parameter estimate	Partial R^2^	Model R^2^	F Value	Variable entered	Parameter estimate	Partial R^2^	Model R^2^	F Value
Non-stress	FLv	8.57	0.02	0.24	9.32**	FLv	5.41	0.0043	0.38	12.42**
	TFC	− 1.43			2.82^n.s^	TFC	− 1.79			7.45**
	Intercept	806.68			205.49***	Anti	5.88			6.34*
						Intercept	250.70			20.72**
Drought stress	TPC	4.39	0.0036	0.62	7.30**	TPC	2.14	0.0001	0.72	4.26*
	FLv	3.42			14.94**	FLv	2.81			24.76***
	TFC	− 1.60			11.04**	TFC	− 0.87			8.11**
	Antho	3.51			3.20^n.s^	Antho	2.53			4.06*
	Intercept	343.22			50.00***	Intercept	144.34			21.62***

n.s, *, ** and *** represent non-significant, significant at *P* < 0.05, *P* < 0.01 and *P* < 0.001, respectively. TPC, total phenol content; TFC, total flavonoid content; FLv, flavanone; Anti, antioxidant activity; Antho, anthocyanin; FFY, forage fresh yield; FDY, forage dry weight.

The results of the Mantel test showed that no significant correlations were found between forage yield traits and geographical data of the collection areas of accessions tested under non-stress environment (r =  − 0.035, *P* = 0.81) and drought stress conditions (r =  − 0.029, *P* = 0.76).

### Genotypic similarities

**Figure 5:**
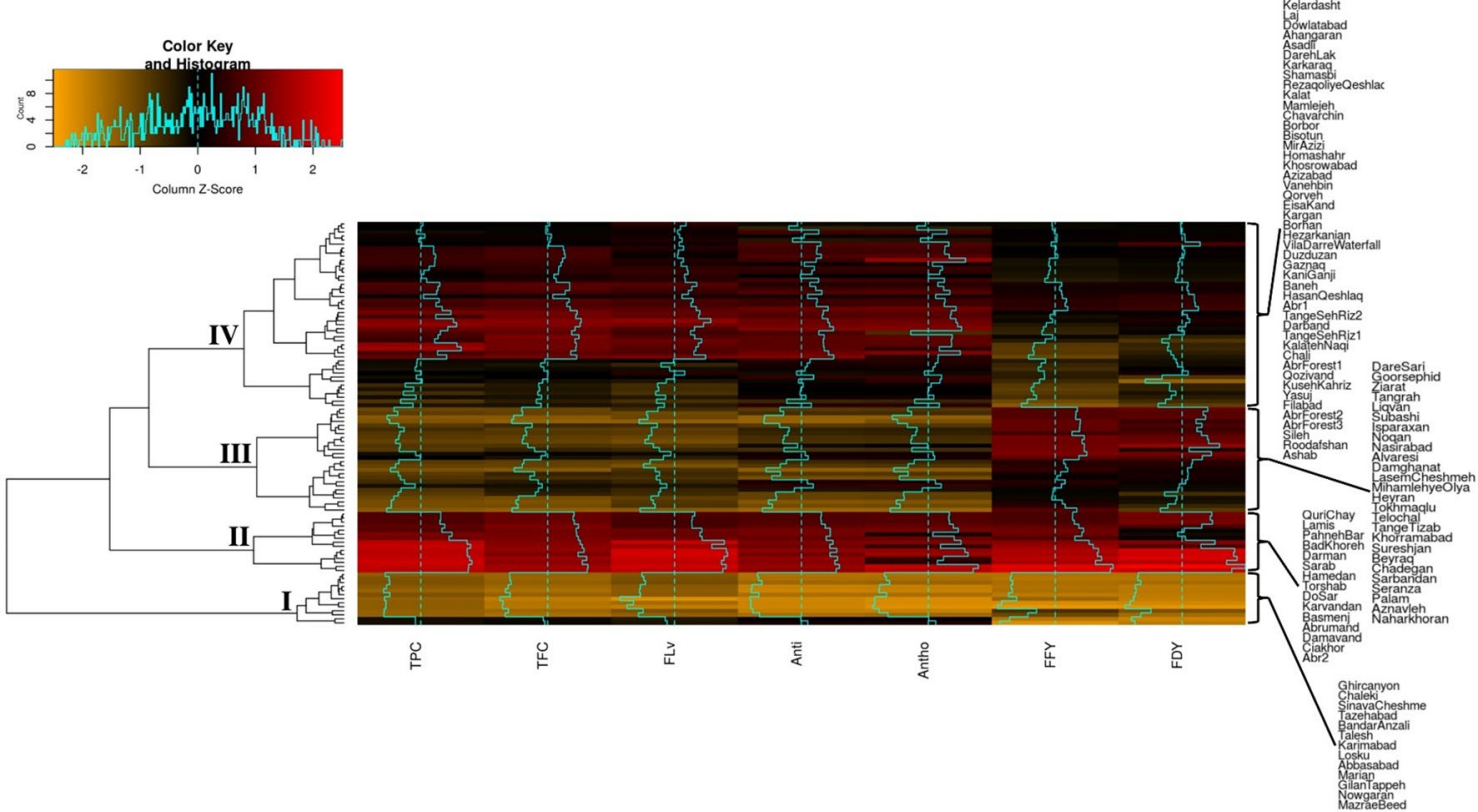
Heat-map and two-dimensional dendrogram for 100 *Poa pratensis* accessions tested for polyphenols content, antioxidant activity and forage yield traits in non-stress treatment. Dendrogram illustrates the relation between accessions (rows) and traits (columns) based on variations in color shades obtained using Z-score. TPC, TFC, FLv, Anti, Antho, FFY and FDY represent vectors of total phenol content, total flavonoid content, flavanone, antioxidant activity, anthocyanin, fresh forage yield and dry forage yield, respectively.

**Figure 6:**
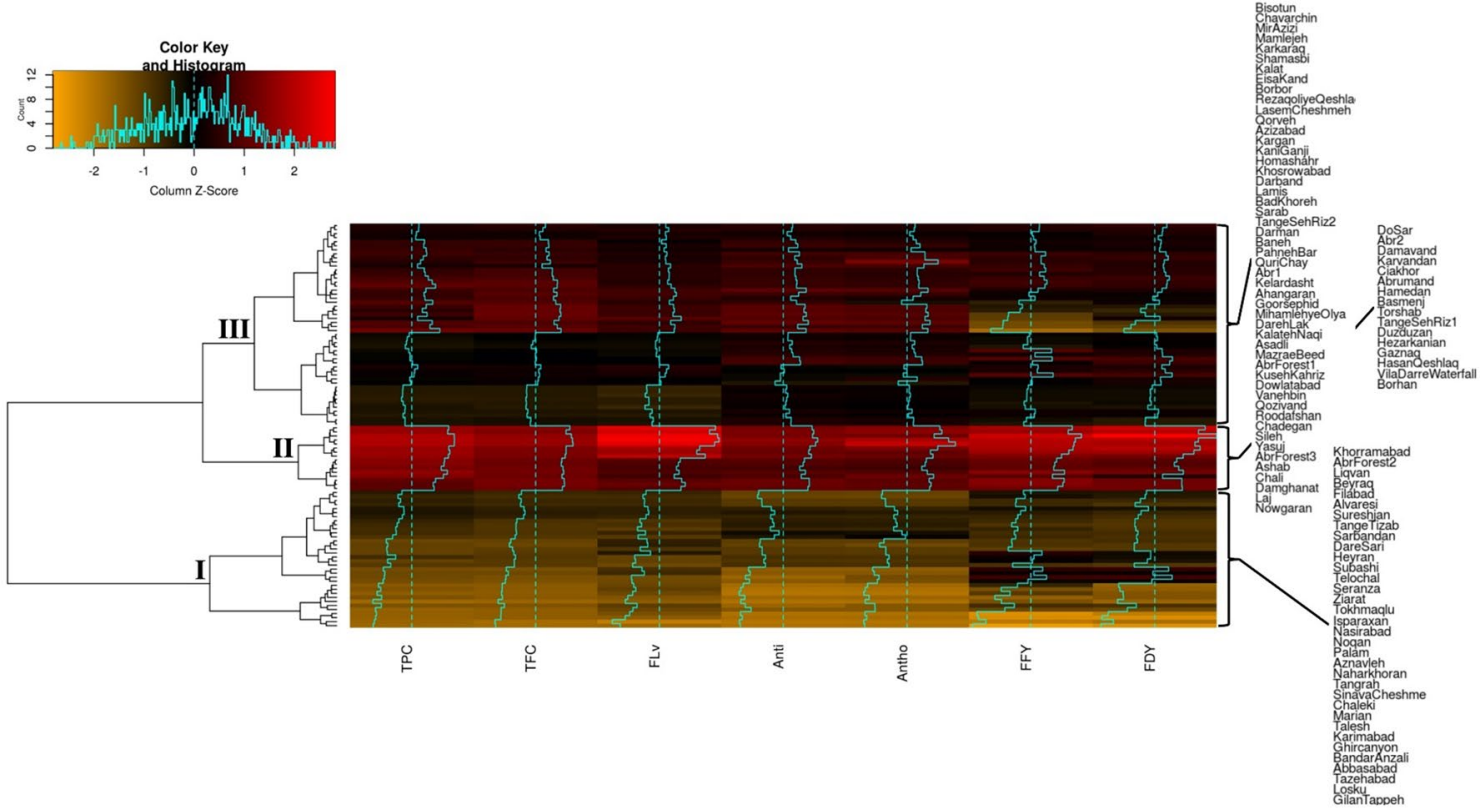
Heat-map and two-dimensional dendrogram for 100 *Poa pratensis* accessions tested for polyphenols content, antioxidant activity and forage yield traits in drought stress. Dendrogram illustrates the relation between accessions (rows) and traits (columns) based on variations in color shades obtained using Z-score. TPC, TFC, FLv, Anti, Antho, FFY and FDY represent vectors of total phenol content, total flavonoid content, flavanone, antioxidant activity, anthocyanin, fresh forage yield and dry forage yield, respectively.

The two-dimensional heatmaps for traits and *P. pratensis* accessions for traits tested under the two moisture conditions are displayed in Figs. 5 and 6. Under non-stress conditions, the accessions were grouped into four distinct clusters based on variations in phytochemical and forage yield traits (Fig. 5). In Cluster I, the accessions showed low values for all traits. Cluster II comprised 15 accessions with high values for all assessed traits. Cluster III harbored 26 accessions showing relatively high fresh and dry forage yields and low TPC, TFC, Anti, FLv, and Antho. Accessions in Cluster IV showed low to moderate forage yield traits and relatively high vales for phytochemical components. The ‘KalatehNaqi’, ‘Chali’, ‘AbrForest1’, ‘Qozivand’, K’usehKahriz’, ‘Yasuj’, ‘Filabad’, ‘AbrForest2’, ‘AbrForest3’, ‘Sileh’, ‘Roodafshan’, and ‘Ashab’ accessions assigned to cluster IV had relatively moderate levels for traits FFY, FDY, TFC, TPC and FLv (Fig. 5).

Under drought stress conditions, the accessions were clustered into three groups (Fig. 6). Clusters I and II showed lowest and highest mean values for all the assessed traits, respectively. Cluster III comprised 50 accessions with relatively moderate phytochemical and forage yield traits (Fig. 6). The result of cluster analysis under non-stress condition showed that except two accessions the majority of accessions classified in cluster I (Fig. 5). ‘Nowgaran’ and ‘MazraeBeed’ accessions in this group had low means for all of the assessed traits under drought stress condition. These two accessions placed in cluster III of drought stress condition (Fig. 6) where most of accessions in this group had moderate phytochemicals and forage yield. Furthermore, of the 15 accessions of the cluster II under non-stress condition (Fig. 5), ‘Hamedan’, ‘Torshab’, ‘DoSar’, ‘Karvandan’, ‘Basmenj’, ‘Abrumand’, ‘Damavand’, ‘Ciakhor’ and ‘Abr2’ showed high values for the traits under drought stress (Fig. 6). Overall, the result of cluster analysis revealed that ‘TangeSehRiz1’, ‘Duzduzan’, ‘Hezarkanian’, ‘Gaznaq’, ‘HassanQeshlaq’, ‘VillaDarreWaterfall’ and ‘Borhan’ with low forage yield under non-stress condition in cluster IV (Fig. 5) presented high forage yield (FFY and FDY) under drought stress condition (Fig. 6). Additionally, ‘QuriChay’, ‘Lamis’, ‘PahnehBar’, ‘BadKhoreh’, ‘Darman’ and ‘Sarab’ with high phytochemical compounds and forage yield under non-stress condition showed moderate values for all traits under drought stress condition (Fig. 6).

## Discussion

In the present study, Kentucky bluegrass accessions collected from a wide range of geographical regions in Iran showed considerable genotypic variation under non-stress and drought stress conditions. Information on the relative extent of genetic variation for economically desirable traits is vital for the breeding of perennial forage grasses^54^. High genetic variation in a germplasm helps in breeding progress under variable environmental conditions. The year × genotype × irrigation regime (Y × G × M) triple interactions were significant for all traits suggesting heterogeneity in response of Kentucky bluegrass to year and moisture regimes. In other words, the assessed accessions show significant variation for phytochemical and forage yield traits across two irrigation regime and years. The means for the tested traits were higher in the second year than in the first year showing variations in environmental conditions of the two years. Heterogeneous responses for antioxidants and yield traits were identified in Kentucky bluegrass in non-stress and drought stress treatments. The Kentucky bluegrass of our study accumulated higher polyphenol compounds under drought stress treatment compared to the non-stress conditions that was in line with results of previous studies in grasses^55–57^. Higher antioxidant activities and phytochemicals identified could be due to either the direct effect of free radical scavenger or indirect stimulation of antioxidant enzymes as a response system^55^. Forage yield of the tested accessions in our study significantly reduced in drought stress treatment. This adverse effect of drought stress on yield traits has been documented in Kentucky bluegrass and other grasses^58–65^.

Our results showed that the GCV and PCV values had small differences in the traits tested. Jalata et al.^66^ in barley and Majidi et al.^67^ in tall fescue showed that the difference between PCV and GCV was smaller for phenological characters than for yield and its components. Estimation of GCV showed that wide genetic diversity existed for TPC, TFC, FLv, Anti, Antho, and fresh and dry forage yield in Kentucky bluegrass. The wide genetic diversity of this perennial grass provides an opportunity to improve forage yield capacity, which is the main objective of breeding programs. Forage yield has shown high variation in other grasses, including *Festuca arundinacea*^67–71^ and *Festuca pratensis*^72–74^. The results of genetic variation analysis in our study revealed that the GCV for agronomic traits was higher for accessions tested under irrigation conditions than under drought conditions, implying that drought stress restricts the phenotypic response of accessions. This shows that selection under non-stress conditions is not guarantee of success under drought stress condition and the necessity of germplasm evaluation under both non-stress and stress conditions for better decision making for selection^71,75^. However, slight differences in GCV estimates for TPC, TFC, and Antho characters in two years under drought conditions suggesting the effects of variable environmental conditions on expression and genotypic variances of these traits and significant genotype by environment interactions that should be taken into consideration in the future breeding program.

In the current study, the highest heritability estimates were obtained for flavanone content, whereas the lowest was estimated for FDY and FFY. However, the estimates of heritability were moderate to high for fresh and dry forage yields, suggesting that phenotypic selection could be successful. In addition, the phytochemical traits showed high heritability, indicating that selection for these traits may be effective for indirect improvement of forage yield. Higher heritability is advantageous for successful selection^76^. The phytochemical components of our study had higher heritability than forage yield traits (FFY and FDY). Therefore, determining the relationship between forage yield and phytochemical composition could lead to the use of an effective criterion for indirect selection under environmental conditions^76^. Indirect selection could be more efficient than direct selection when indirect traits show higher heritability and correlation with yield traits^75^. Additionally, lower heritability of the FFY and FDY traits under drought stress compared to non-stress condition could be due to environmental variances of two moisture regimes^77^. Blum^75^ showed that heritability for yield is higher and the rate of genetic advance through selection is usually greater in an optimal environment.

The results of the correlation analysis revealed that phytochemical composition significantly correlated under irrigation regimes. Information on the covariance of traits is useful for predicting how the selection pressure exerted on one trait will cause changes in other traits^78,79^. The correlation between phenol contents and antioxidant capacity has been identified in other grasses^80–82^. The results of this study indicated that phytochemicals and forage yield traits did correlate in both conditions, suggesting the protective role of these compounds against drought stress. Phenolic compounds scavenge ROS, catalyze oxygenation reactions through the formation of metallic complexes, and inhibit the activities of oxidizing enzymes^83–86^. The correlation of forage yield and phytochemical traits identified under drought stress conditions in the present study shows the possibility of successful selection for both high forage yield and polyphenol content. The results of analysis of the interrelations of traits indicated that phytochemical traits had a higher contribution to the observed variation in fresh and dry forage yield under drought stress conditions, which could be due to the positive role of these compounds in coping with the adverse effects of drought stress and thus improving performance^82^. In this study, the results of the Mantel test showed no significant correlation between geographical and forage yield traits suggesting no isolation by distance (IBD) between *P.pratensis* accesions. One possible explanation is that populations that were not significantly different from one another were from a similar source population rather than connected by gene flow^87^. These results are in agreement with the results of Dennhardt et al.^87^ in Kentucky bluegrass.

The results of the clustering analysis in our study indicated that accessions collected from neighboring regions grouped in the same clade. Nonetheless, accessions of the same geographic origin necessarily not positioned in the same cluster. Cluster analysis results indicated that the 100 Kentucky bluegrass accessions could be divided into distinct groups based on their response to drought stress. In the Chai et al.^88^ study, the Kentucky bluegrass varieties divided into four drought tolerant groups. In our study, ‘Hamedan’, ‘Torshab’, ‘DoSar’, ‘Karvandan’, ‘Basmenj’, ‘Abrumand’, ‘Damavand’, ‘Ciakhor’ and ‘Abr2’ presented high mean values for all of the assessed traits under both non-stress and drought stress conditions. Such genotypes showing superiority under both non-stress and stress conditions possibly accumulate favorable genes for the tested traits and can be involved in pre-breeding programs for forage breeding programs. Although ‘TangeSehRiz1’, ‘Duzduzan’, ‘Hezarkanian’, ‘Gaznaq’, ‘HasanQeshlaq’, ‘VilaDarreWaterfall’ and ‘Borhan’ showed low forage yield traits (FFY and FDY) under non-stress condition they presented high forage yield under drought stress, possibly because of their higher potential to cope with the adverse effects of drought stress through their efficient antioxidant systems. Several accessions including ‘Gilantapeh’, ‘Losku’, ‘Karimabad’, ‘Abbasabad’ showed susceptibility to drought stress condition.

## Conclusions

The results showed that drought stress increased antioxidant activity and polyphenolic compounds, such as total phenol and flavonoids, in Kentucky bluegrass accessions. However, a significant reduction in forage yield was observed under drought stress conditions. High PCV, GCV, and heritability estimates for the tested traits, especially under drought stress, indicated the possibility of improvement of Kentucky bluegrass in terms of forage yield and phytochemical traits through selective breeding. The significant associations of forage yield with phytochemical traits identified through regression and correlation analysis helped in the simultaneous selection of both forage yield and phytochemical traits. As a result, wide variation observed in accessions tested helps to select good candidates for cross-breeding programs to produce drought-tolerant varieties containing higher phytochemical and forage yield traits.

## Supplementary Information

Supplementary Legends.

Supplementary Table S1.

Supplementary Table S2.

## Data Availability

The datasets generated during and/or analyzed during the current study are available from the corresponding author on reasonable request.

## Abbreviations

Antho: Anthocyanin
Anti: Antioxidant activity
DW: Dry weight
FDY: Forage dry yield
FFY: Forage fresh yield
FLv: Flavanone
GCV: Genotypic coefficients of variation
IBD: Isolation by distance
PCV: Phenotypic coefficients of variation
TFC: Total flavonoid content
TPC: Total phenol content
