# Supplementary material for: Assessing the potential of native ecotypes of Poa pratensis L. for forage yield and phytochemical compositions under water deficit conditions
Source: Sci Rep. 2022 Jan 21;12:1121. doi: 10.1038/s41598-022-05024-1 (PMC8782833; doi:10.1038/s41598-022-05024-1)
Supplement: Supplementary file 1 — Supplementary Legends. [file 41598_2022_5024_MOESM1_ESM.docx]

**Supplementary Information**

**Supplementary Table S1.** Geographic information of original sites of assessed one-hundred genotypes collected from different parts of Iran.

**Supplementary Table S2.** Mean values of studied traits measured from one-hundred genotypes of Kentucky Bluegrass evaluated in non-stress and drought stress environments during years 2018 and 2019.
