# Supplementary material for: Assessing the potential of native ecotypes of Poa pratensis L. for forage yield and phytochemical compositions under water deficit conditions
Source: Sci Rep. 2022 Jan 21;12:1121. doi: 10.1038/s41598-022-05024-1 (PMC8782833; doi:10.1038/s41598-022-05024-1)
Supplement: Supplementary file 3 — Supplementary Table S2. [file 41598_2022_5024_MOESM3_ESM.docx]

| **Supplemental Table S2** Mean values of studied traits measured from one-hundred genotypes of Kentucky Bluegrass evaluated in irrigated and non-irrigated environments during years 2018 and 2019. | | | | | | | | | | |
| --- | --- | --- | --- | --- | --- | --- | --- | --- | --- | --- |
| G | Irrigated | | | | |  | Non-irrigated | | | |
|  | 2018 | | 2019 | | |  | 2018 | | 2019 | |
|  | TPC | ± SE | TPC | | ± SE |  | TPC | ± SE | TPC | ± SE |
| Abbasabad | 44.07 | ± 0.22 | 50.39 | | ± 0.06 |  | 47.20 | ± 0.10 | 58.82 | ± 0.34 |
| Abr1Forest1 | 84.18 | ± 0.07 | 119.20 | | ± 0.08 |  | 101.42 | ± 0.07 | 128.41 | ± 0.26 |
| Abr2Forest2 | 61.24 | ± 0.02 | 78.07 | | ± 0.00 |  | 79.75 | ± 0.56 | 106.94 | ± 0.79 |
| Abr3Forest3 | 110.28 | ± 13.71 | 110.31 | | ± 13.72 |  | 115.23 | ± 0.53 | 129.96 | ± 0.30 |
| Abr1 | 52.86 | ± 0.10 | 84.32 | | ± 0.01 |  | 59.34 | ± 0.45 | 88.74 | ± 0.00 |
| Abr2 | 52.44 | ± 0.02 | 68.72 | | ± 0.01 |  | 66.19 | ± 0.47 | 92.13 | ± 0.79 |
| Abrumand | 98.17 | ± 1.37 | 124.66 | | ± 0.7 |  | 123.88 | ± 7.44 | 123.48 | ± 8.93 |
| Ahangaran | 70.25 | ± 1.06 | 83.86 | | ± 0.10 |  | 68.64 | ± 1.07 | 97.60 | ± 0.07 |
| Alvaresi | 53.08 | ± 0.07 | 63.30 | | ± 0.71 |  | 63.35 | ± 0.23 | 96.74 | ± 0.25 |
| Asadli | 61.81 | ± 1.45 | 83.21 | | ± 0.00 |  | 73.48 | ± 0.26 | 98.14 | ± 1.44 |
| Ashab | 59.42 | ± 0.01 | 76.57 | | ± 0.02 |  | 65.01 | ± 0.50 | 97.33 | ± 0.01 |
| Azizabad | 77.21 | ± 0.05 | 95.21 | | ± 0.05 |  | 82.25 | ± 0.00 | 119.06 | ± 0.12 |
| Aznãvleh | 48.24 | ± 0.68 | 54.61 | | ± 1.45 |  | 52.78 | ± 0.49 | 69.68 | ± 0.47 |
| BadKhoreh | 83.97 | ± 0.11 | 100.59 | | ± 0.07 |  | 94.43 | ± 0.08 | 121.54 | ± 1.09 |
| BandarehAnzali | 42.18 | ± 0.23 | 48.75 | | ± 0.08 |  | 45.77 | ± 0.06 | 58.45 | ± 0.36 |
| Baneh | 81.63 | ± 0.02 | 92.75 | | ± 0.06 |  | 83.09 | ± 0.05 | 103.33 | ± 0.51 |
| Basmenj | 92.85 | ± 0.27 | 126.51 | | ± 0.14 |  | 113.55 | ± 0.05 | 133.07 | ± 0.02 |
| Beyraq | 60.95 | ± 0.01 | 78.24 | | ± 0.28 |  | 59.91 | ± 0.54 | 89.81 | ± 0.66 |
| Bisotun | 68.99 | ± 0.03 | 79.76 | | ± 0.62 |  | 78.53 | ± 0.35 | 110.44 | ± 0.71 |
| Borbor | 69.56 | ± 0.05 | 83.14 | | ± 0.02 |  | 84.09 | ± 1.21 | 113.38 | ± 0.38 |
| Borhan | 76.46 | ± 0.05 | 117.56 | | ± 0.46 |  | 94.06 | ± 2.29 | 124.61 | ± 1.36 |
| Chadegan | 51.56 | ± 0.05 | 66.10 | | ± 0.84 |  | 64.81 | ± 0.09 | 92.54 | ± 0.50 |
| Chaleki | 46.43 | ± 0.05 | 52.91 | | ± 0.55 |  | 49.20 | ± 0.51 | 65.37 | ± 1.18 |
| Chali | 57.11 | ± 0.00 | 84.60 | | ± 0.02 |  | 66.11 | ± 0.23 | 95.42 | ± 0.18 |
| Chavarchin | 76.66 | ± 0.10 | 84.10 | | ± 0.01 |  | 78.44 | ± 0.25 | 110.04 | ± 0.65 |
| Ciakhor | 96.64 | ± 0.03 | 124.64 | | ± 0.07 |  | 118.71 | ± 1.15 | 139.41 | ± 1.55 |
| Damavand | 98.03 | ± 1.93 | 128.24 | | ± 0.06 |  | 118.30 | ± 0.36 | 139.81 | ± 0.15 |
| Damghanat | 49.80 | ± 1.76 | 65.07 | | ± 0.03 |  | 65.88 | ± 0.49 | 92.21 | ± 0.09 |
| Darband | 75.21 | ± 0.05 | 98.29 | | ± 0.00 |  | 91.92 | ± 0.09 | 107.16 | ± 0.06 |
| DareSari | 48.61 | ± 0.75 | 58.24 | | ± 0.08 |  | 56.90 | ± 0.43 | 87.00 | ± 0.26 |
| Darman | 80.14 | ± 0.02 | 100.17 | | ± 2.39 |  | 92.47 | ± 1.49 | 112.55 | ± 1.52 |
| DarrehLak | 66.77 | ± 0.79 | 82.18 | | ± 0.03 |  | 71.91 | ± 1.95 | 97.34 | ± 0.79 |
| DoSar | 97.59 | ± 0.02 | 126.70 | | ± 0.14 |  | 112.31 | ± 1.56 | 134.85 | ± 0.99 |
| Dowlatabad | 63.33 | ± 1.20 | 78.13 | | ± 0.09 |  | 76.20 | ± 1.15 | 103.46 | ± 1.06 |
| Duzduzan | 84.45 | ± 0.41 | 118.40 | | ± 0.75 |  | 106.83 | ± 0.26 | 125.69 | ± 2.41 |
| EisaKand | 77.49 | ± 0.08 | 93.77 | | ± 0.11 |  | 85.43 | ± 1.57 | 118.79 | ± 1.29 |
| Filabad | 52.53 | ± 0.04 | 71.51 | | ± 0.03 |  | 63.03 | ± 0.11 | 96.56 | ± 0.80 |
| Gaznaq | 83.46 | ± 0.05 | 105.91 | | ± 0.20 |  | 102.99 | ± 1.41 | 133.88 | ± 0.31 |
| Ghircanyon1 | 40.91 | ± 0.05 | 52.54 | | ± 0.07 |  | 51.32 | ± 0.06 | 64.37 | ± 0.44 |
| GilanTappeh | 40.12 | ± 0.08 | 51.26 | | ± 0.47 |  | 44.80 | ± 0.07 | 55.04 | ± 0.39 |
| Goorsephid | 50.20 | ± 0.06 | 62.44 | | ± 0.03 |  | 67.28 | ± 0.19 | 97.28 | ± 0.77 |
| Hamashahr | 77.60 | ± 0.06 | 99.34 | | ± 1.50 |  | 99.81 | ± 0.61 | 121.73 | ± 1.21 |
| Hamedan | 81.73 | ± 0.68 | 109.48 | | ± 1.55 |  | 118.18 | ± 0.10 | 131.19 | ± 0.04 |
| HasanQeshlaq | 80.31 | ± 0.31 | 88.70 | | ± 0.63 |  | 93.55 | ± 0.58 | 128.00 | ± 0.03 |
| Heyran | 51.84 | ± 0.08 | 62.59 | | ± 1.48 |  | 58.66 | ± 0.18 | 86.14 | ± 0.65 |
| Hezarkanian | 72.96 | ± 1.55 | 97.86 | | ± 0.42 |  | 109.92 | ± 0.69 | 134.63 | ± 0.21 |
| Isparaxan | 53.06 | ± 2.30 | 62.61 | | ± 0.00 |  | 56.87 | ± 0.19 | 86.85 | ± 0.69 |
| Kalat | 75.66 | ± 0.0 | 93.89 | | ± 0.13 |  | 83.88 | ± 0.08 | 119.38 | ± 0.60 |
| KalatehNaqi | 63.72 | ± 0.0 | 78.62 | | ± 0.68 |  | 72.25 | ± 0.37 | 99.41 | ± 0.33 |
| KaniGanji | 75.71 | ± 2.6 | 102.30 | | ± 0.03 |  | 91.14 | ± 0.90 | 123.93 | ± 0.23 |
| Kargan | 78.40 | ± 0.34 | 102.44 | | ± 1.08 |  | 83.41 | ± 0.21 | 123.96 | ± 0.86 |
| Karimabad | 42.79 | ± 0.86 | 49.69 | | ± 0.66 |  | 42.61 | ± 0.05 | 56.52 | ± 0.18 |
| Karkaraq | 74.84 | ± 0.0 | 92.79 | | ± 0.08 |  | 84.58 | ± 1.57 | 115.42 | ± 0.20 |
| Karvandan | 96.73 | ± 0.08 | 122.20 | | ± 0.77 |  | 119.20 | ± 0.66 | 139.21 | ± 0.66 |
| Kelardasht | 67.72 | ± 1.04 | 83.41 | | ± 0.04 |  | 71.71 | ± 0.18 | 98.45 | ± 0.59 |
| Khorramabad | 53.20 | ± 0.06 | 63.70 | | ± 0.06 |  | 62.51 | ± 0.59 | 93.38 | ± 0.56 |
| Khosrowabad | 77.91 | ± 0.08 | 96.34 | | ± 0.27 |  | 82.98 | ± 0.02 | 119.70 | ± 0.35 |
| KusehKahriz | 62.93 | ± 0.97 | 78.16 | | ± 0.02 |  | 75.52 | ± 1.24 | 105.20 | ± 1.02 |
| Laj | 65.25 | ± 0.01 | 83.04 | | ± 0.02 |  | 66.39 | ± 0.38 | 95.72 | ± 0.85 |
| Lamis | 75.84 | ± 0.02 | 102.68 | | ± 0.30 |  | 83.51 | ± 1.61 | 117.21 | ± 0.39 |
| LasemCheshmeh | 61.70 | ± 0.02 | 78.60 | | ± 0.09 |  | 78.05 | ± 1.23 | 108.20 | ± 0.11 |
| Liqvan | 48.80 | ± 0.51 | 65.19 | | ± 0.17 |  | 61.04 | ± 0.34 | 92.20 | ± 1.72 |
| Losku | 41.19 | ± 0.23 | 53.52 | | ± 0.18 |  | 43.64 | ± 0.06 | 58.42 | ± 0.69 |
| Mamlejeh | 77.02 | ± 0.01 | 92.17 | | ± 0.01 |  | 74.99 | ± 0.31 | 107.62 | ± 1.13 |
| Marian | 40.79 | ± 0.03 | 51.19 | | ± 0.07 |  | 48.28 | ± 0.14 | 59.06 | ± 0.89 |
| MazraeBeed | 61.23 | ± 0.02 | 78.61 | | ± 0.10 |  | 75.93 | ± 0.87 | 100.56 | ± 0.42 |
| MihamlehyeOlya | 62.19 | ± 0.03 | 85.20 | | ± 0.33 |  | 66.39 | ± 0.21 | 96.04 | ± 0.90 |
| MirAzizi | 74.72 | ± 0.04 | 82.29 | | ± 0.08 |  | 75.52 | ± 1.07 | 108.50 | ± 0.50 |
| Naharkhoran | 40.31 | ± 0.0 | 55.36 | | ± 0.35 |  | 51.18 | ± 0.56 | 70.22 | ± 0.88 |
| Nasirabad | 49.96 | ± 0.05 | 59.49 | | ± 0.08 |  | 52.52 | ± 0.33 | 75.35 | ± 0.10 |
| Noqan | 50.54 | ± 0.03 | 63.56 | | ± 0.05 |  | 56.12 | ± 0.19 | 77.93 | ± 0.48 |
| Nowgaran | 61.21 | ± 2.10 | 78.56 | | ± 0.34 |  | 66.34 | ± 0.30 | 95.49 | ± 1.10 |
| PahnehBar | 76.83 | ± 0.01 | 100.50 | | ± 0.31 |  | 93.92 | ± 1.55 | 121.20 | ± 0.03 |
| Palam | 48.64 | ± 0.04 | 54.26 | | ± 0.60 |  | 51.47 | ± 0.54 | 68.23 | ± 0.22 |
| Qorveh | 77.94 | ± 0.13 | 98.24 | | ± 1.42 |  | 82.80 | ± 1.58 | 116.70 | ± 0.63 |
| Qozivand | 65.44 | ± 0.02 | 83.12 | | ± 0.01 |  | 73.65 | ± 0.73 | 99.83 | ± 0.27 |
| QuriChay | 76.11 | ± 0.37 | 102.54 | | ± 0.49 |  | 88.15 | ± 1.54 | 122.32 | ± 0.63 |
| RezaqoliyeQeshlaq | 76.37 | ± 0.06 | 94.37 | | ± 0.06 |  | 83.48 | ± 0.13 | 108.75 | ± 0.00 |
| Roodafshan | 51.44 | ± 0.82 | 65.67 | | ± 0.96 |  | 66.15 | ± 0.76 | 91.84 | ± 0.23 |
| Sarab | 79.66 | ± 0.60 | 116.19 | | ± 0.67 |  | 94.45 | ± 0.41 | 108.93 | ± 0.33 |
| Sarbandan | 49.01 | ± 0.45 | 64.46 | | ± 0.10 |  | 57.95 | ± 0.96 | 88.63 | ± 0.05 |
| Seranza | 48.34 | ± 0.48 | 58.22 | | ± 0.06 |  | 53.48 | ± 0.24 | 74.53 | ± 0.29 |
| Shamasbi | 72.94 | ± 0.90 | 94.71 | | ± 0.05 |  | 85.02 | ± 0.73 | 113.45 | ± 0.05 |
| TangeSehRiz1 | 73.04 | ± 0.98 | 114.58 | | ± 0.03 |  | 105.75 | ± 0.72 | 132.33 | ± 0.63 |
| Sileh | 60.29 | ± 0.07 | 80.56 | | ± 1.34 |  | 65.37 | ± 0.09 | 91.75 | ± 0.80 |
| SinavaCheshme | 43.27 | ± 0.34 | 56.91 | | ± 0.80 |  | 48.37 | ± 0.47 | 61.78 | ± 0.19 |
| Subashi | 52.63 | ± 0.06 | 69.84 | | ± 0.57 |  | 56.04 | ± 0.11 | 81.68 | ± 0.71 |
| Sureshjan | 55.41 | ± 1.55 | 67.56 | | ± 0.01 |  | 62.37 | ± 0.41 | 94.37 | ± 1.01 |
| Talesh | 47.13 | ± 0.55 | 53.39 | | ± 0.43 |  | 49.62 | ± 0.47 | 66.60 | ± 0.10 |
| TangeSehRiz2 | 88.46 | ± 0.55 | 121.38 | | ± 0.63 |  | 86.67 | ± 1.54 | 108.94 | ± 0.31 |
| TangeTizab | 48.97 | ± 0.41 | 64.69 | | ± 4.38 |  | 58.62 | ± 0.25 | 91.34 | ± 0.26 |
| Tangrah | 42.14 | ± 0.07 | 57.96 | | ± 0.90 |  | 51.20 | ± 0.04 | 68.19 | ± 0.25 |
| Tazehabad | 44.98 | ± 0.01 | 50.68 | | ± 0.14 |  | 45.96 | ± 0.27 | 58.62 | ± 0.60 |
| Telochal | 46.11 | ± 0.05 | 55.37 | | ± 0.32 |  | 50.51 | ± 0.64 | 74.76 | ± 1.06 |
| Tokhmaqlu | 49.29 | ± 0.53 | 60.00 | | ± 0.94 |  | 50.76 | ± 0.20 | 76.63 | ± 0.35 |
| Torshab | 95.99 | ± 0.01 | 123.01 | | ± 0.05 |  | 111.96 | ± 2.40 | 127.04 | ± 1.10 |
| Vanehbin | 72.99 | ± 0.03 | 79.12 | | ± 0.02 |  | 73.67 | ± 0.54 | 97.64 | ± 0.00 |
| Vila Darre Waterfall | 81.53 | ± 1.21 | 116.53 | | ± 0.49 |  | 95.82 | ± 0.58 | 121.17 | ± 1.37 |
| Yasuj | 61.11 | ± 0.05 | 88.97 | | ± 0.68 |  | 63.03 | ± 0.69 | 92.10 | ± 0.29 |
| Ziarat | 41.08 | ± 0.07 | 55.21 | | ± 0.05 |  | 50.44 | ± 0.25 | 75.85 | ± 0.58 |
| G | Irrigated | | | | |  | Non-irrigated | | | |
|  | 2018 | | 2019 | | |  | 2018 | | 2019 | |
|  | TFC | ± SE | TFC | | ± SE |  | TFC | ± SE | TFC | ± SE |
| Abbasabad | 92.92 | ± 0.41 | 109.24 | | ± 0.71 |  | 113.57 | ± 0.14 | 134.62 | ± 0.46 |
| Abr1Forest1 | 264.80 | ± 0.97 | 324.95 | | ± 0.38 |  | 316.04 | ± 0.30 | 354.27 | ± 0.50 |
| Abr2Forest2 | 193.24 | ± 0.29 | 225.79 | | ± 0.05 |  | 247.78 | ± 0.43 | 280.14 | ± 0.57 |
| Abr3Forest3 | 323.32 | ± 42.56 | 323.53 | | ± 40.88 |  | 330.79 | ± 0.64 | 374.41 | ± 0.96 |
| Abr1 | 187.45 | ± 0.62 | 209.53 | | ± 0.46 |  | 167.24 | ± 0.29 | 206.09 | ± 0.96 |
| Abr2 | 182.02 | ± 0.48 | 205.69 | | ± 0.08 |  | 192.78 | ± 0.56 | 234.23 | ± 0.55 |
| Abrumand | 282.32 | ± 0.44 | 361.31 | | ± 0.44 |  | 353.05 | ± 29.99 | 352.45 | ± 30.07 |
| Ahangaran | 219.47 | ± 0.00 | 239.12 | | ± 0.13 |  | 231.99 | ± 2.97 | 255.86 | ± 0.66 |
| Alvaresi | 156.77 | ± 0.58 | 178.52 | | ± 0.03 |  | 188.02 | ± 0.74 | 229.14 | ± 0.70 |
| Asadli | 218.08 | ± 1.05 | 234.72 | | ± 0.00 |  | 236.33 | ± 0.31 | 260.11 | ± 0.71 |
| Ashab | 189.06 | ± 0.15 | 215.74 | | ± 0.19 |  | 191.89 | ± 0.31 | 231.95 | ± 1.74 |
| Azizabad | 244.13 | ± 0.3 | 287.21 | | ± 0.58 |  | 293.79 | ± 0.86 | 314.39 | ± 0.07 |
| Aznãvleh | 134.07 | ± 0.67 | 149.61 | | ± 0.44 |  | 138.65 | ± 0.60 | 161.27 | ± 0.72 |
| BadKhoreh | 264.82 | ± 0.53 | 336.11 | | ± 1.14 |  | 303.48 | ± 0.37 | 329.74 | ± 0.00 |
| BandarehAnzali | 97.52 | ± 2.26 | 120.62 | | ± 0.33 |  | 117.26 | ± 1.73 | 132.15 | ± 1.95 |
| Baneh | 264.42 | ± 0.12 | 338.79 | | ± 1.16 |  | 314.99 | ± 0.57 | 340.34 | ± 0.50 |
| Basmenj | 277.80 | ± 0.43 | 320.57 | | ± 0.89 |  | 328.23 | ± 0.72 | 370.88 | ± 0.46 |
| Beyraq | 190.12 | ± 0.58 | 220.51 | | ± 0.20 |  | 168.31 | ± 0.47 | 216.93 | ± 0.09 |
| Bisotun | 227.99 | ± 0.77 | 243.94 | | ± 0.33 |  | 261.40 | ± 1.21 | 298.73 | ± 0.32 |
| Borbor | 232.30 | ± 0.35 | 263.31 | | ± 0.28 |  | 268.59 | ± 0.71 | 302.37 | ± 0.65 |
| Borhan | 267.82 | ± 0.14 | 325.89 | | ± 0.70 |  | 320.15 | ± 0.27 | 359.51 | ± 0.76 |
| Chadegan | 181.38 | ± 0.10 | 208.99 | | ± 0.64 |  | 193.11 | ± 0.30 | 234.14 | ± 0.34 |
| Chaleki | 115.30 | ± 0.45 | 125.68 | | ± 0.05 |  | 121.70 | ± 0.13 | 134.96 | ± 0.60 |
| Chali | 187.98 | ± 0.40 | 214.93 | | ± 0.91 |  | 199.08 | ± 0.20 | 238.25 | ± 0.32 |
| Chavarchin | 234.16 | ± 0.32 | 263.44 | | ± 0.33 |  | 252.76 | ± 0.02 | 290.66 | ± 0.13 |
| Ciakhor | 280.22 | ± 0.55 | 370.81 | | ± 1.15 |  | 354.64 | ± 0.23 | 399.50 | ± 1.07 |
| Damavand | 274.97 | ± 0.12 | 345.64 | | ± 3.05 |  | 350.48 | ± 0.90 | 392.44 | ± 0.13 |
| Damghanat | 178.47 | ± 0.35 | 192.24 | | ± 1.04 |  | 195.01 | ± 0.83 | 236.02 | ± 0.06 |
| Darband | 245.97 | ± 0.55 | 314.48 | | ± 0.41 |  | 308.97 | ± 0.27 | 332.93 | ± 0.02 |
| DareSari | 140.97 | ± 0.53 | 162.49 | | ± 1.93 |  | 161.37 | ± 0.35 | 192.32 | ± 0.92 |
| Darman | 264.25 | ± 1.06 | 324.45 | | ± 1.34 |  | 311.72 | ± 0.30 | 357.51 | ± 0.20 |
| DarrehLak | 212.06 | ± 0.27 | 232.80 | | ± 1.96 |  | 235.70 | ± 0.25 | 261.80 | ± 1.77 |
| DoSar | 272.51 | ± 1.38 | 348.14 | | ± 1.50 |  | 346.86 | ± 0.99 | 385.08 | ± 0.24 |
| Dowlatabad | 196.14 | ± 0.18 | 233.68 | | ± 0.30 |  | 240.70 | ± 0.36 | 278.62 | ± 0.62 |
| Duzduzan | 269.89 | ± 0.83 | 339.53 | | ± 0.03 |  | 319.49 | ± 1.43 | 364.18 | ± 0.73 |
| EisaKand | 247.89 | ± 0.30 | 292.02 | | ± 0.77 |  | 275.04 | ± 0.84 | 311.29 | ± 1.64 |
| Filabad | 184.44 | ± 0.60 | 207.64 | | ± 0.57 |  | 181.72 | ± 0.58 | 228.00 | ± 0.92 |
| Gaznaq | 263.08 | ± 0.73 | 319.98 | | ± 0.00 |  | 315.85 | ± 0.30 | 360.96 | ± 0.63 |
| Ghircanyon1 | 114.40 | ± 0.45 | 125.00 | | ± 0.84 |  | 123.71 | ± 0.22 | 148.44 | ± 2.60 |
| GilanTappeh | 97.28 | ± 0.27 | 119.58 | | ± 0.28 |  | 96.88 | ± 1.05 | 113.69 | ± 0.31 |
| Goorsephid | 144.98 | ± 0.76 | 163.00 | | ± 1.03 |  | 224.39 | ± 1.49 | 248.55 | ± 0.34 |
| Hamashahr | 242.68 | ± 0.31 | 311.96 | | ± 0.46 |  | 310.08 | ± 0.84 | 332.72 | ± 0.80 |
| Hamedan | 270.11 | ± 1.66 | 329.91 | | ± 0.30 |  | 330.42 | ± 0.68 | 375.14 | ± 0.81 |
| HasanQeshlaq | 256.98 | ± 1.28 | 325.64 | | ± 0.30 |  | 323.08 | ± 0.57 | 369.19 | ± 1.00 |
| Heyran | 148.91 | ± 0.18 | 165.43 | | ± 0.57 |  | 161.14 | ± 0.50 | 195.74 | ± 0.31 |
| Hezarkanian | 258.21 | ± 0.65 | 328.76 | | ± 0.69 |  | 323.30 | ± 0.53 | 365.27 | ± 0.16 |
| Isparaxan | 156.58 | ± 0.74 | 172.22 | | ± 0.08 |  | 160.49 | ± 0.73 | 189.83 | ± 0.31 |
| Kalat | 240.60 | ± 0.98 | 277.41 | | ± 0.30 |  | 262.83 | ± 1.00 | 301.35 | ± 0.49 |
| KalatehNaqi | 194.86 | ± 0.33 | 229.35 | | ± 0.43 |  | 231.15 | ± 1.10 | 258.57 | ± 1.33 |
| KaniGanji | 248.50 | ± 0.45 | 316.52 | | ± 0.36 |  | 310.48 | ± 0.26 | 334.57 | ± 0.98 |
| Kargan | 243.80 | ± 0.45 | 313.48 | | ± 1.41 |  | 299.01 | ± 0.86 | 316.79 | ± 0.59 |
| Karimabad | 92.53 | ± 0.03 | 96.74 | | ± 0.52 |  | 101.45 | ± 0.55 | 126.47 | ± 0.16 |
| Karkaraq | 239.68 | ± 0.48 | 281.91 | | ± 0.57 |  | 270.83 | ± 0.27 | 310.29 | ± 0.01 |
| Karvandan | 270.88 | ± 0.15 | 359.28 | | ± 1.5 |  | 351.91 | ± 0.86 | 398.10 | ± 1.38 |
| Kelardasht | 213.77 | ± 0.75 | 235.40 | | ± 0.53 |  | 231.78 | ± 1.21 | 257.69 | ± 0.22 |
| Khorramabad | 163.98 | ± 0.28 | 182.79 | | ± 0.15 |  | 169.35 | ± 0.31 | 222.58 | ± 0.17 |
| Khosrowabad | 248.77 | ± 0.58 | 305.94 | | ± 0.22 |  | 299.83 | ± 1.41 | 318.67 | ± 0.41 |
| KusehKahriz | 210.98 | ± 0.07 | 228.19 | | ± 1.02 |  | 238.20 | ± 0.42 | 275.14 | ± 0.52 |
| Laj | 195.12 | ± 0.08 | 235.32 | | ± 1.29 |  | 211.25 | ± 0.27 | 245.90 | ± 0.92 |
| Lamis | 249.08 | ± 2.13 | 323.57 | | ± 1.86 |  | 300.90 | ± 0.66 | 316.60 | ± 0.21 |
| LasemCheshmeh | 202.85 | ± 0.37 | 226.29 | | ± 1.18 |  | 250.66 | ± 0.99 | 285.95 | ± 0.52 |
| Liqvan | 166.89 | ± 0.28 | 190.85 | | ± 0.39 |  | 168.79 | ± 0.52 | 221.70 | ± 0.14 |
| Losku | 94.34 | ± 0.39 | 111.13 | | ± 0.70 |  | 96.23 | ± 0.63 | 109.80 | ± 0.46 |
| Mamlejeh | 236.21 | ± 0.73 | 273.43 | | ± 0.27 |  | 243.78 | ± 0.78 | 279.00 | ± 0.16 |
| Marian | 110.62 | ± 0.00 | 122.54 | | ± 0.31 |  | 116.83 | ± 0.45 | 134.93 | ± 0.10 |
| MazraeBeed | 201.02 | ± 0.36 | 222.89 | | ± 0.06 |  | 227.95 | ± 1.05 | 260.70 | ± 0.64 |
| MihamlehyeOlya | 189.83 | ± 2.95 | 214.93 | | ± 0.91 |  | 215.27 | ± 0.05 | 246.02 | ± 0.77 |
| MirAzizi | 230.52 | ± 0.21 | 253.80 | | ± 0.27 |  | 242.92 | ± 0.49 | 274.45 | ± 0.16 |
| Naharkhoran | 118.10 | ± 0.36 | 133.79 | | ± 0.05 |  | 127.38 | ± 0.28 | 155.40 | ± 0.80 |
| Nasirabad | 143.20 | ± 0.59 | 162.89 | | ± 1.78 |  | 153.06 | ± 1.35 | 188.18 | ± 0.34 |
| Noqan | 147.24 | ± 0.08 | 164.95 | | ± 0.07 |  | 154.93 | ± 2.09 | 186.83 | ± 5.59 |
| Nowgaran | 192.27 | ± 0.71 | 223.87 | | ± 0.11 |  | 210.12 | ± 0.37 | 243.73 | ± 0.70 |
| PahnehBar | 256.67 | ± 0.13 | 322.26 | | ± 0.19 |  | 300.09 | ± 0.82 | 321.01 | ± 1.92 |
| Palam | 125.51 | ± 0.22 | 147.92 | | ± 0.30 |  | 127.59 | ± 0.96 | 157.64 | ± 2.84 |
| Qorveh | 246.45 | ± 0.50 | 305.75 | | ± 0.19 |  | 295.63 | ± 0.16 | 313.89 | ± 0.72 |
| Qozivand | 197.54 | ± 0.34 | 242.92 | | ± 0.23 |  | 241.11 | ± 0.23 | 265.31 | ± 0.46 |
| QuriChay | 254.76 | ± 0.66 | 323.16 | | ± 0.54 |  | 302.10 | ± 0.86 | 341.31 | ± 3.16 |
| RezaqoliyeQeshlaq | 244.22 | ± 0.65 | 287.00 | | ± 1.07 |  | 281.85 | ± 0.30 | 313.00 | ± 0.14 |
| Roodafshan | 179.23 | ± 0.07 | 200.54 | | ± 0.35 |  | 192.26 | ± 0.08 | 237.12 | ± 0.34 |
| Sarab | 266.02 | ± 0.00 | 325.18 | | ± 0.30 |  | 313.51 | ± 0.36 | 353.69 | ± 0.39 |
| Sarbandan | 177.38 | ± 0.48 | 197.85 | | ± 0.46 |  | 158.91 | ± 0.95 | 195.14 | ± 0.29 |
| Seranza | 130.04 | ± 0.74 | 156.26 | | ± 0.80 |  | 139.26 | ± 0.12 | 168.33 | ± 0.17 |
| Shamasbi | 237.22 | ± 0.59 | 289.37 | | ± 0.51 |  | 271.30 | ± 0.55 | 312.61 | ± 0.34 |
| TangeSehRiz1 | 265.90 | ± 0.59 | 325.48 | | ± 0.45 |  | 318.72 | ± 0.30 | 352.57 | ± 0.69 |
| Sileh | 189.00 | ± 0.15 | 218.30 | | ± 0.43 |  | 193.29 | ± 0.37 | 236.16 | ± 1.14 |
| SinavaCheshme | 116.35 | ± 0.38 | 131.08 | | ± 0.35 |  | 121.03 | ± 0.10 | 135.63 | ± 0.92 |
| Subashi | 150.54 | ± 0.77 | 172.18 | | ± 0.03 |  | 158.19 | ± 0.96 | 189.47 | ± 1.25 |
| Sureshjan | 181.74 | ± 0.77 | 207.72 | | ± 0.34 |  | 180.73 | ± 1.17 | 226.49 | ± 0.37 |
| Talesh | 114.99 | ± 0.01 | 127.05 | | ± 0.04 |  | 122.01 | ± 0.14 | 136.11 | ± 0.88 |
| TangeSehRiz2 | 263.58 | ± 1.45 | 323.84 | | ± 0.02 |  | 310.72 | ± 0.84 | 352.31 | ± 0.23 |
| TangeTizab | 174.89 | ± 0.76 | 195.26 | | ± 0.59 |  | 162.55 | ± 0.18 | 197.00 | ± 1.72 |
| Tangrah | 119.10 | ± 0.72 | 131.73 | | ± 1.11 |  | 124.09 | ± 0.38 | 154.76 | ± 2.05 |
| Tazehabad | 110.55 | ± 1.26 | 121.80 | | ± 0.77 |  | 113.50 | ± 0.11 | 133.35 | ± 0.62 |
| Telochal | 127.32 | ± 0.29 | 136.64 | | ± 0.69 |  | 139.67 | ± 0.77 | 177.40 | ± 0.96 |
| Tokhmaqlu | 143.10 | ± 0.32 | 162.77 | | ± 3.98 |  | 152.59 | ± 2.20 | 186.26 | ± 1.10 |
| Torshab | 268.99 | ± 0.12 | 340.71 | | ± 1.06 |  | 321.42 | ± 0.27 | 354.87 | ± 0.61 |
| Vanehbin | 226.18 | ± 0.52 | 240.21 | | ± 0.48 |  | 237.72 | ± 0.01 | 272.93 | ± 0.23 |
| Vila Darre Waterfall | 274.28 | ± 0.27 | 341.33 | | ± 0.03 |  | 325.30 | ± 0.44 | 368.80 | ± 0.15 |
| Yasuj | 191.19 | ± 0.65 | 221.89 | | ± 0.27 |  | 189.91 | ± 0.78 | 233.73 | ± 0.16 |
| Ziarat | 125.10 | ± 0.15 | 135.71 | | ± 0.24 |  | 150.91 | ± 0.89 | 178.55 | ± 2.95 |
| G | Irrigated | | | | |  | Non-irrigated | | | |
|  | 2018 | | 2019 | | |  | 2018 | | 2019 | |
|  | FLv | ± SE | FLv | | ± SE |  | FLv | ± SE | FLv | ± SE |
| Abbasabad | 26.57 | ± 0.51 | 39.46 | | ± 0.04 |  | 44.72 | ± 0.06 | 56.35 | ± 0.70 |
| Abr1Forest1 | 81.09 | ± 0.40 | 99.06 | | ± 0.64 |  | 89.66 | ± 0.45 | 100.78 | ± 0.69 |
| Abr2Forest2 | 58.98 | ± 0.48 | 75.32 | | ± 0.00 |  | 84.38 | ± 0.03 | 94.90 | ± 0.52 |
| Abr3Forest3 | 109.66 | ± 4.33 | 109.59 | | ± 5.20 |  | 118.15 | ± 0.92 | 143.30 | ± 0.57 |
| Abr1 | 52.42 | ± 0.01 | 64.24 | | ± 0.31 |  | 53.70 | ± 0.50 | 74.04 | ± 0.15 |
| Abr2 | 50.68 | ± 0.35 | 61.18 | | ± 0.18 |  | 65.10 | ± 0.14 | 74.63 | ± 0.56 |
| Abrumand | 98.57 | ± 0.33 | 122.50 | | ± 0.04 |  | 121.50 | ± 4.22 | 120.20 | ± 4.24 |
| Ahangaran | 63.00 | ± 0.45 | 87.09 | | ± 0.37 |  | 73.59 | ± 0.38 | 82.66 | ± 0.11 |
| Alvaresi | 48.58 | ± 0.65 | 59.66 | | ± 1.19 |  | 64.51 | ± 0.81 | 74.58 | ± 0.78 |
| Asadli | 69.07 | ± 0.03 | 85.18 | | ± 1.32 |  | 73.41 | ± 0.10 | 86.62 | ± 0.33 |
| Ashab | 54.99 | ± 0.22 | 70.91 | | ± 0.78 |  | 55.77 | ± 0.11 | 75.89 | ± 0.13 |
| Azizabad | 76.33 | ± 0.29 | 87.85 | | ± 0.58 |  | 83.80 | ± 0.05 | 96.70 | ± 0.36 |
| Aznãvleh | 41.40 | ± 0.03 | 69.61 | | ± 0.07 |  | 60.28 | ± 0.27 | 70.24 | ± 0.14 |
| BadKhoreh | 78.33 | ± 0.43 | 91.61 | | ± 0.39 |  | 81.04 | ± 0.51 | 99.64 | ± 1.11 |
| BandarehAnzali | 37.86 | ± 0.26 | 48.20 | | ± 0.19 |  | 47.65 | ± 0.43 | 59.80 | ± 0.06 |
| Baneh | 81.78 | ± 0.33 | 92.34 | | ± 0.29 |  | 93.38 | ± 0.06 | 110.99 | ± 0.25 |
| Basmenj | 97.32 | ± 0.09 | 129.23 | | ± 0.69 |  | 117.05 | ± 0.57 | 135.75 | ± 0.19 |
| Beyraq | 57.48 | ± 0.33 | 72.14 | | ± 0.79 |  | 55.12 | ± 0.09 | 75.23 | ± 0.25 |
| Bisotun | 64.76 | ± 0.50 | 93.22 | | ± 0.04 |  | 79.69 | ± 0.45 | 93.80 | ± 0.74 |
| Borbor | 68.43 | ± 1.01 | 95.16 | | ± 0.06 |  | 74.92 | ± 0.00 | 97.70 | ± 0.31 |
| Borhan | 82.77 | ± 0.18 | 99.67 | | ± 0.3 |  | 95.31 | ± 0.59 | 108.66 | ± 0.85 |
| Chadegan | 50.49 | ± 0.03 | 69.02 | | ± 0.36 |  | 59.29 | ± 0.90 | 76.32 | ± 0.38 |
| Chaleki | 37.06 | ± 0.37 | 56.00 | | ± 0.76 |  | 53.39 | ± 0.16 | 59.80 | ± 0.46 |
| Chali | 53.58 | ± 0.13 | 66.75 | | ± 0.00 |  | 67.85 | ± 0.18 | 78.19 | ± 0.46 |
| Chavarchin | 69.06 | ± 0.51 | 92.93 | | ± 0.51 |  | 83.96 | ± 0.15 | 95.42 | ± 1.03 |
| Ciakhor | 107.41 | ± 1.33 | 117.05 | | ± 0.30 |  | 125.74 | ± 0.90 | 145.77 | ± 0.49 |
| Damavand | 98.22 | ± 0.29 | 118.73 | | ± 0.73 |  | 134.25 | ± 1.36 | 143.54 | ± 0.08 |
| Damghanat | 51.33 | ± 0.68 | 66.56 | | ± 0.08 |  | 65.26 | ± 0.06 | 76.45 | ± 0.40 |
| Darband | 78.89 | ± 0.09 | 94.03 | | ± 0.31 |  | 89.55 | ± 0.96 | 105.14 | ± 0.02 |
| DareSari | 42.30 | ± 0.85 | 73.23 | | ± 0.13 |  | 50.98 | ± 0.34 | 65.62 | ± 1.08 |
| Darman | 87.66 | ± 0.17 | 98.01 | | ± 0.41 |  | 86.78 | ± 0.41 | 101.28 | ± 1.50 |
| DarrehLak | 66.85 | ± 0.05 | 82.45 | | ± 0.44 |  | 73.92 | ± 0.23 | 81.65 | ± 1.46 |
| DoSar | 88.82 | ± 0.44 | 112.67 | | ± 1.33 |  | 131.48 | ± 1.08 | 141.79 | ± 0.15 |
| Dowlatabad | 64.66 | ± 0.22 | 82.37 | | ± 0.43 |  | 77.82 | ± 0.15 | 94.56 | ± 0.04 |
| Duzduzan | 85.22 | ± 0.01 | 103.15 | | ± 0.22 |  | 87.04 | ± 0.99 | 107.89 | ± 1.11 |
| EisaKand | 77.38 | ± 0.12 | 91.16 | | ± 0.69 |  | 81.71 | ± 0.55 | 94.12 | ± 1.84 |
| Filabad | 50.74 | ± 0.04 | 64.13 | | ± 0.03 |  | 61.90 | ± 0.05 | 75.83 | ± 0.38 |
| Gaznaq | 80.19 | ± 0.58 | 93.63 | | ± 0.30 |  | 88.79 | ± 0.37 | 101.74 | ± 0.29 |
| Ghircanyon1 | 38.32 | ± 0.10 | 51.65 | | ± 0.47 |  | 52.87 | ± 0.26 | 62.88 | ± 0.73 |
| GilanTappeh | 36.92 | ± 0.17 | 47.31 | | ± 0.11 |  | 37.10 | ± 0.18 | 44.93 | ± 0.42 |
| Goorsephid | 45.41 | ± 0.34 | 52.07 | | ± 0.07 |  | 71.35 | ± 0.73 | 81.80 | ± 0.27 |
| Hamashahr | 81.45 | ± 0.02 | 96.06 | | ± 0.64 |  | 86.54 | ± 0.28 | 97.83 | ± 0.08 |
| Hamedan | 84.64 | ± 0.09 | 101.39 | | ± 0.15 |  | 109.07 | ± 0.76 | 126.63 | ± 0.53 |
| HasanQeshlaq | 86.11 | ± 1.03 | 99.23 | | ± 0.49 |  | 95.32 | ± 0.42 | 110.08 | ± 0.05 |
| Heyran | 46.95 | ± 0.34 | 56.08 | | ± 0.09 |  | 58.90 | ± 0.05 | 73.78 | ± 0.87 |
| Hezarkanian | 83.30 | ± 0.73 | 97.28 | | ± 1.26 |  | 89.30 | ± 0.64 | 110.80 | ± 0.53 |
| Isparaxan | 47.22 | ± 0.19 | 58.45 | | ± 1.47 |  | 48.48 | ± 0.12 | 62.51 | ± 0.40 |
| Kalat | 71.62 | ± 0.46 | 84.57 | | ± 1.18 |  | 76.82 | ± 0.08 | 93.04 | ± 0.30 |
| KalatehNaqi | 63.86 | ± 0.12 | 76.44 | | ± 0.53 |  | 73.09 | ± 0.36 | 81.35 | ± 1.23 |
| KaniGanji | 77.36 | ± 0.59 | 90.23 | | ± 0.39 |  | 85.89 | ± 0.09 | 91.29 | ± 0.73 |
| Kargan | 81.06 | ± 0.43 | 95.69 | | ± 0.65 |  | 84.69 | ± 0.18 | 95.44 | ± 0.59 |
| Karimabad | 26.65 | ± 0.02 | 28.14 | | ± 0.00 |  | 38.92 | ± 0.10 | 53.31 | ± 0.60 |
| Karkaraq | 71.20 | ± 0.70 | 85.34 | | ± 0.11 |  | 75.54 | ± 3.10 | 89.61 | ± 0.54 |
| Karvandan | 103.40 | ± 0.45 | 121.35 | | ± 0.50 |  | 131.26 | ± 1.14 | 149.30 | ± 1.55 |
| Kelardasht | 71.83 | ± 1.12 | 88.53 | | ± 1.19 |  | 74.99 | ± 0.23 | 82.46 | ± 0.51 |
| Khorramabad | 49.93 | ± 0.47 | 62.99 | | ± 0.28 |  | 58.28 | ± 0.04 | 75.36 | ± 0.45 |
| Khosrowabad | 78.41 | ± 0.23 | 91.17 | | ± 1.13 |  | 89.20 | ± 0.05 | 102.38 | ± 0.94 |
| KusehKahriz | 63.60 | ± 0.33 | 77.63 | | ± 0.28 |  | 79.50 | ± 0.47 | 95.46 | ± 1.08 |
| Laj | 67.12 | ± 0.31 | 84.43 | | ± 0.43 |  | 67.36 | ± 0.05 | 81.01 | ± 0.46 |
| Lamis | 79.33 | ± 0.06 | 91.12 | | ± 0.06 |  | 86.85 | ± 0.51 | 101.94 | ± 0.17 |
| LasemCheshmeh | 59.63 | ± 0.02 | 76.56 | | ± 1.13 |  | 86.20 | ± 1.15 | 96.88 | ± 0.58 |
| Liqvan | 54.78 | ± 0.14 | 59.69 | | ± 0.03 |  | 55.70 | ± 0.89 | 75.26 | ± 0.13 |
| Losku | 34.32 | ± 0.49 | 42.32 | | ± 0.4 |  | 29.11 | ± 0.36 | 36.82 | ± 0.09 |
| Mamlejeh | 70.09 | ± 0.65 | 82.45 | | ± 0.48 |  | 81.66 | ± 0.07 | 92.84 | ± 0.34 |
| Marian | 39.01 | ± 0.93 | 52.92 | | ± 0.16 |  | 48.41 | ± 1.11 | 58.48 | ± 0.14 |
| MazraeBeed | 57.62 | ± 0.08 | 72.62 | | ± 0.65 |  | 73.96 | ± 0.15 | 86.96 | ± 1.11 |
| MihamlehyeOlya | 54.87 | ± 0.10 | 69.33 | | ± 0.16 |  | 69.18 | ± 0.57 | 83.75 | ± 0.48 |
| MirAzizi | 64.95 | ± 0.46 | 94.23 | | ± 2.57 |  | 81.13 | ± 0.43 | 91.56 | ± 0.23 |
| Naharkhoran | 39.45 | ± 0.07 | 47.48 | | ± 0.3 |  | 47.45 | ± 0.05 | 57.05 | ± 0.36 |
| Nasirabad | 43.28 | ± 0.08 | 51.54 | | ± 0.6 |  | 44.12 | ± 0.46 | 68.56 | ± 0.44 |
| Noqan | 46.63 | ± 0.21 | 54.00 | | ± 0.94 |  | 46.35 | ± 0.05 | 60.56 | ± 0.45 |
| Nowgaran | 56.64 | ± 0.08 | 70.63 | | ± 0.28 |  | 67.16 | ± 0.24 | 78.64 | ± 0.07 |
| PahnehBar | 81.90 | ± 0.74 | 87.76 | | ± 0.41 |  | 82.35 | ± 0.51 | 100.95 | ± 0.13 |
| Palam | 39.21 | ± 0.33 | 68.87 | | ± 0.13 |  | 57.18 | ± 0.13 | 68.53 | ± 0.27 |
| Qorveh | 79.67 | ± 0.22 | 91.81 | | ± 1.17 |  | 83.94 | ± 0.17 | 94.94 | ± 0.80 |
| Qozivand | 70.16 | ± 1.15 | 88.19 | | ± 1.02 |  | 76.07 | ± 0.39 | 87.73 | ± 1.36 |
| QuriChay | 81.87 | ± 0.00 | 92.36 | | ± 0.35 |  | 89.82 | ± 0.60 | 102.04 | ± 0.41 |
| RezaqoliyeQeshlaq | 64.51 | ± 1.37 | 82.40 | | ± 0.20 |  | 81.13 | ± 0.40 | 92.35 | ± 0.90 |
| Roodafshan | 50.31 | ± 0.05 | 62.89 | | ± 0.10 |  | 62.91 | ± 0.74 | 72.04 | ± 0.63 |
| Sarab | 84.82 | ± 0.53 | 97.75 | | ± 0.74 |  | 88.89 | ± 0.74 | 100.32 | ± 1.35 |
| Sarbandan | 50.87 | ± 0.76 | 65.69 | | ± 0.71 |  | 52.51 | ± 0.18 | 72.36 | ± 1.09 |
| Seranza | 39.46 | ± 0.21 | 65.02 | | ± 0.32 |  | 64.95 | ± 0.33 | 72.53 | ± 0.40 |
| Shamasbi | 69.57 | ± 0.54 | 78.43 | | ± 1.00 |  | 79.17 | ± 0.6 | 95.14 | ± 2.45 |
| TangeSehRiz1 | 88.58 | ± 0.23 | 103.01 | | ± 1.73 |  | 91.57 | ± 0.12 | 107.34 | ± 0.19 |
| Sileh | 56.53 | ± 0.22 | 71.23 | | ± 1.51 |  | 57.55 | ± 0.41 | 74.76 | ± 0.26 |
| SinavaCheshme | 38.26 | ± 1.02 | 57.30 | | ± 0.05 |  | 48.49 | ± 0.07 | 60.53 | ± 0.06 |
| Subashi | 47.12 | ± 0.44 | 58.10 | | ± 0.36 |  | 53.28 | ± 0.02 | 64.72 | ± 1.25 |
| Sureshjan | 50.65 | ± 1.18 | 68.53 | | ± 0.06 |  | 59.81 | ± 0.41 | 73.85 | ± 0.01 |
| Talesh | 36.30 | ± 0.00 | 51.15 | | ± 1.25 |  | 42.49 | ± 1.74 | 54.81 | ± 0.19 |
| TangeSehRiz2 | 77.06 | ± 0.55 | 98.99 | | ± 0.78 |  | 90.02 | ± 0.61 | 100.63 | ± 0.52 |
| TangeTizab | 53.63 | ± 0.32 | 64.48 | | ± 0.29 |  | 50.98 | ± 0.03 | 70.68 | ± 1.28 |
| Tangrah | 36.15 | ± 0.18 | 49.32 | | ± 0.18 |  | 43.91 | ± 0.38 | 61.62 | ± 2.31 |
| Tazehabad | 37.79 | ± 0.16 | 49.78 | | ± 0.27 |  | 43.27 | ± 0.64 | 48.82 | ± 0.91 |
| Telochal | 45.10 | ± 0.29 | 66.46 | | ± 0.18 |  | 61.87 | ± 0.93 | 73.96 | ± 0.49 |
| Tokhmaqlu | 42.32 | ± 0.34 | 50.24 | | ± 0.49 |  | 64.92 | ± 0.61 | 77.93 | ± 1.94 |
| Torshab | 102.22 | ± 0.00 | 119.32 | | ± 0.45 |  | 95.59 | ± 0.50 | 106.94 | ± 0.20 |
| Vanehbin | 63.20 | ± 0.56 | 90.65 | | ± 0.10 |  | 71.89 | ± 0.99 | 89.51 | ± 0.59 |
| Vila Darre Waterfall | 96.19 | ± 0.00 | 103.72 | | ± 0.98 |  | 100.74 | ± 0.59 | 111.83 | ± 0.35 |
| Yasuj | 56.62 | ± 0.05 | 72.35 | | ± 0.29 |  | 64.81 | ± 0.19 | 74.68 | ± 0.38 |
| Ziarat | 40.05 | ± 0.04 | 59.32 | | ± 0.10 |  | 53.83 | ± 0.14 | 68.28 | ± 0.16 |
| G | Irrigated | | | | |  | Non-irrigated | | | |
|  | 2018 | | 2019 | | |  | 2018 | | 2019 | |
|  | Anti | ± SE | Anti | | ± SE |  | Anti | ± SE | Anti | ± SE |
| Abbasabad | 30.04 | ± 0.14 | 35.54 | | ± 0.06 |  | 34.02 | ± 0.14 | 39.89 | ± 1.06 |
| Abr1Forest1 | 59.95 | ± 0.09 | 74.34 | | ± 0.31 |  | 71.53 | ± 0.25 | 79.74 | ± 1.36 |
| Abr2Forest2 | 54.89 | ± 0.12 | 62.06 | | ± 1.45 |  | 61.55 | ± 0.13 | 60.39 | ± 0.35 |
| Abr3Forest3 | 73.33 | ± 3.37 | 72.92 | | ± 3.44 |  | 74.34 | ± 0.07 | 85.96 | ± 0.15 |
| Abr1 | 50.76 | ± 0.10 | 62.24 | | ± 0.03 |  | 43.16 | ± 0.04 | 51.08 | ± 0.11 |
| Abr2 | 49.96 | ± 0.72 | 62.20 | | ± 1.31 |  | 54.25 | ± 0.05 | 73.12 | ± 0.50 |
| Abrumand | 71.07 | ± 0.33 | 76.32 | | ± 0.00 |  | 77.70 | ± 6.61 | 78.24 | ± 6.48 |
| Ahangaran | 54.11 | ± 0.19 | 61.96 | | ± 0.70 |  | 58.52 | ± 0.49 | 71.32 | ± 0.12 |
| Alvaresi | 51.19 | ± 0.39 | 58.70 | | ± 0.94 |  | 48.94 | ± 1.04 | 65.39 | ± 0.41 |
| Asadli | 48.47 | ± 0.08 | 56.64 | | ± 0.11 |  | 64.28 | ± 0.05 | 79.36 | ± 0.51 |
| Ashab | 53.00 | ± 0.75 | 69.84 | | ± 0.09 |  | 54.93 | ± 0.05 | 71.37 | ± 0.96 |
| Azizabad | 58.84 | ± 0.06 | 73.91 | | ± 0.21 |  | 62.20 | ± 0.25 | 76.01 | ± 0.05 |
| Aznãvleh | 38.03 | ± 0.89 | 47.97 | | ± 0.11 |  | 38.87 | ± 0.42 | 46.49 | ± 0.13 |
| BadKhoreh | 62.94 | ± 0.22 | 72.90 | | ± 0.03 |  | 63.26 | ± 0.12 | 76.77 | ± 0.36 |
| BandarehAnzali | 29.75 | ± 0.18 | 36.53 | | ± 0.05 |  | 41.50 | ± 0.03 | 44.97 | ± 0.09 |
| Baneh | 62.60 | ± 0.21 | 72.28 | | ± 0.09 |  | 68.57 | ± 0.12 | 79.16 | ± 0.22 |
| Basmenj | 63.84 | ± 0.02 | 76.51 | | ± 0.41 |  | 72.66 | ± 0.08 | 84.86 | ± 0.56 |
| Beyraq | 52.07 | ± 0.04 | 72.55 | | ± 0.39 |  | 44.51 | ± 1.21 | 54.82 | ± 0.28 |
| Bisotun | 57.01 | ± 0.07 | 55.57 | | ± 0.28 |  | 61.46 | ± 0.18 | 74.08 | ± 0.11 |
| Borbor | 57.80 | ± 0.73 | 65.51 | | ± 0.03 |  | 61.98 | ± 0.07 | 75.89 | ± 1.06 |
| Borhan | 61.46 | ± 0.53 | 72.59 | | ± 0.02 |  | 73.06 | ± 0.47 | 80.37 | ± 0.56 |
| Chadegan | 47.67 | ± 1.82 | 59.14 | | ± 0.75 |  | 55.44 | ± 0.52 | 71.66 | ± 0.16 |
| Chaleki | 31.60 | ± 0.10 | 41.50 | | ± 0.03 |  | 35.09 | ± 0.12 | 39.23 | ± 0.09 |
| Chali | 51.40 | ± 0.99 | 67.53 | | ± 0.32 |  | 58.25 | ± 0.05 | 73.64 | ± 0.41 |
| Chavarchin | 57.87 | ± 1.79 | 67.80 | | ± 0.01 |  | 60.65 | ± 0.35 | 73.86 | ± 0.11 |
| Ciakhor | 71.51 | ± 0.00 | 77.71 | | ± 0.04 |  | 73.44 | ± 0.13 | 89.06 | ± 0.16 |
| Damavand | 69.18 | ± 0.09 | 75.72 | | ± 0.62 |  | 73.29 | ± 0.00 | 85.61 | ± 0.07 |
| Damghanat | 45.04 | ± 0.18 | 51.93 | | ± 0.05 |  | 55.94 | ± 0.45 | 71.46 | ± 0.01 |
| Darband | 62.98 | ± 0.22 | 76.00 | | ± 0.87 |  | 65.09 | ± 0.06 | 77.43 | ± 0.10 |
| DareSari | 44.35 | ± 0.51 | 48.86 | | ± 0.31 |  | 49.63 | ± 0.09 | 59.55 | ± 1.04 |
| Darman | 65.70 | ± 0.11 | 74.85 | | ± 0.17 |  | 70.61 | ± 0.40 | 80.96 | ± 0.56 |
| DarrehLak | 57.05 | ± 0.24 | 74.65 | | ± 0.86 |  | 59.36 | ± 0.82 | 75.05 | ± 1.00 |
| DoSar | 64.16 | ± 0.06 | 75.99 | | ± 0.09 |  | 74.47 | ± 1.50 | 83.91 | ± 0.05 |
| Dowlatabad | 56.91 | ± 0.11 | 72.90 | | ± 0.17 |  | 53.64 | ± 0.28 | 67.40 | ± 0.35 |
| Duzduzan | 66.28 | ± 0.12 | 73.66 | | ± 0.37 |  | 67.00 | ± 0.12 | 76.72 | ± 0.11 |
| EisaKand | 59.62 | ± 0.24 | 67.81 | | ± 0.11 |  | 67.08 | ± 0.17 | 76.24 | ± 0.69 |
| Filabad | 49.19 | ± 0.55 | 65.89 | | ± 0.31 |  | 47.97 | ± 0.84 | 62.30 | ± 2.11 |
| Gaznaq | 67.39 | ± 0.18 | 76.24 | | ± 0.32 |  | 67.46 | ± 1.50 | 78.07 | ± 0.39 |
| Ghircanyon1 | 31.51 | ± 0.09 | 40.61 | | ± 0.02 |  | 35.54 | ± 0.55 | 44.05 | ± 0.24 |
| GilanTappeh | 31.60 | ± 0.10 | 42.34 | | ± 0.10 |  | 32.71 | ± 0.59 | 41.09 | ± 0.00 |
| Goorsephid | 46.26 | ± 0.18 | 50.42 | | ± 0.02 |  | 60.87 | ± 0.50 | 74.17 | ± 0.41 |
| Hamashahr | 58.86 | ± 0.09 | 62.50 | | ± 0.10 |  | 62.94 | ± 0.02 | 77.08 | ± 0.11 |
| Hamedan | 63.25 | ± 0.09 | 73.51 | | ± 0.02 |  | 72.86 | ± 0.10 | 81.57 | ± 0.23 |
| HasanQeshlaq | 62.26 | ± 0.18 | 71.84 | | ± 0.06 |  | 71.44 | ± 0.38 | 83.56 | ± 0.07 |
| Heyran | 46.68 | ± 0.15 | 52.59 | | ± 0.14 |  | 55.05 | ± 0.19 | 64.46 | ± 1.16 |
| Hezarkanian | 61.91 | ± 0.05 | 71.15 | | ± 0.14 |  | 71.50 | ± 0.94 | 79.03 | ± 0.21 |
| Isparaxan | 47.78 | ± 0.29 | 52.85 | | ± 0.45 |  | 48.95 | ± 0.05 | 54.60 | ± 0.35 |
| Kalat | 57.60 | ± 0.28 | 69.74 | | ± 2.00 |  | 57.75 | ± 0.20 | 72.84 | ± 0.33 |
| KalatehNaqi | 54.20 | ± 0.01 | 67.27 | | ± 0.34 |  | 63.44 | ± 0.45 | 72.45 | ± 0.28 |
| KaniGanji | 61.42 | ± 0.19 | 68.38 | | ± 0.14 |  | 63.80 | ± 0.26 | 76.73 | ± 0.02 |
| Kargan | 63.56 | ± 0.10 | 75.00 | | ± 1.67 |  | 63.21 | ± 0.10 | 76.14 | ± 0.11 |
| Karimabad | 29.61 | ± 0.18 | 35.47 | | ± 0.23 |  | 32.41 | ± 1.06 | 41.91 | ± 0.02 |
| Karkaraq | 58.74 | ± 0.24 | 69.36 | | ± 0.05 |  | 64.60 | ± 1.23 | 74.56 | ± 0.22 |
| Karvandan | 66.90 | ± 1.05 | 79.85 | | ± 0.90 |  | 76.15 | ± 0.15 | 88.85 | ± 0.05 |
| Kelardasht | 50.12 | ± 0.27 | 59.69 | | ± 0.29 |  | 59.20 | ± 0.76 | 70.83 | ± 2.04 |
| Khorramabad | 37.99 | ± 0.17 | 47.51 | | ± 0.05 |  | 44.22 | ± 0.14 | 51.19 | ± 0.05 |
| Khosrowabad | 59.71 | ± 0.15 | 67.17 | | ± 0.02 |  | 71.17 | ± 0.42 | 80.23 | ± 0.30 |
| KusehKahriz | 55.49 | ± 0.14 | 69.45 | | ± 0.70 |  | 57.74 | ± 0.33 | 67.81 | ± 1.08 |
| Laj | 49.13 | ± 0.10 | 52.53 | | ± 0.25 |  | 61.46 | ± 0.21 | 72.37 | ± 0.41 |
| Lamis | 61.59 | ± 0.13 | 70.08 | | ± 0.29 |  | 64.46 | ± 0.11 | 68.68 | ± 0.80 |
| LasemCheshmeh | 54.92 | ± 0.22 | 62.10 | | ± 0.65 |  | 71.80 | ± 0.46 | 74.54 | ± 0.63 |
| Liqvan | 43.49 | ± 0.07 | 49.29 | | ± 0.05 |  | 39.78 | ± 0.11 | 59.34 | ± 0.12 |
| Losku | 30.21 | ± 0.06 | 36.00 | | ± 0.17 |  | 32.74 | ± 0.14 | 40.02 | ± 0.14 |
| Mamlejeh | 53.21 | ± 0.34 | 63.03 | | ± 0.21 |  | 61.23 | ± 0.27 | 71.93 | ± 0.11 |
| Marian | 31.31 | ± 0.05 | 36.41 | | ± 1.35 |  | 34.08 | ± 0.09 | 38.51 | ± 0.01 |
| MazraeBeed | 52.35 | ± 0.05 | 67.15 | | ± 0.03 |  | 66.29 | ± 0.63 | 78.39 | ± 0.47 |
| MihamlehyeOlya | 50.98 | ± 0.66 | 68.87 | | ± 0.78 |  | 58.28 | ± 0.38 | 73.71 | ± 0.15 |
| MirAzizi | 60.64 | ± 1.12 | 68.99 | | ± 0.49 |  | 58.35 | ± 0.80 | 70.63 | ± 0.40 |
| Naharkhoran | 34.02 | ± 0.11 | 42.68 | | ± 0.17 |  | 35.38 | ± 0.03 | 44.39 | ± 0.06 |
| Nasirabad | 45.89 | ± 0.18 | 50.15 | | ± 0.34 |  | 46.87 | ± 0.18 | 54.65 | ± 0.56 |
| Noqan | 46.13 | ± 0.00 | 50.97 | | ± 0.01 |  | 49.02 | ± 0.43 | 54.41 | ± 0.37 |
| Nowgaran | 48.97 | ± 1.62 | 63.89 | | ± 0.20 |  | 61.14 | ± 0.89 | 72.26 | ± 0.86 |
| PahnehBar | 61.93 | ± 0.05 | 70.34 | | ± 0.06 |  | 72.71 | ± 0.25 | 81.18 | ± 0.12 |
| Palam | 37.85 | ± 0.16 | 44.58 | | ± 0.28 |  | 38.61 | ± 0.25 | 44.90 | ± 0.19 |
| Qorveh | 60.47 | ± 0.19 | 67.78 | | ± 0.18 |  | 62.82 | ± 0.10 | 75.45 | ± 0.55 |
| Qozivand | 51.59 | ± 0.24 | 57.26 | | ± 0.16 |  | 53.26 | ± 0.22 | 61.51 | ± 0.26 |
| QuriChay | 62.17 | ± 0.93 | 70.08 | | ± 0.29 |  | 65.10 | ± 1.82 | 76.17 | ± 0.18 |
| RezaqoliyeQeshlaq | 59.80 | ± 0.37 | 72.30 | | ± 0.14 |  | 64.25 | ± 0.19 | 76.63 | ± 0.08 |
| Roodafshan | 44.41 | ± 0.11 | 55.21 | | ± 1.13 |  | 55.54 | ± 0.35 | 73.01 | ± 0.15 |
| Sarab | 67.79 | ± 0.13 | 72.81 | | ± 0.38 |  | 70.19 | ± 0.02 | 79.88 | ± 0.24 |
| Sarbandan | 39.88 | ± 0.92 | 49.32 | | ± 0.13 |  | 51.10 | ± 0.11 | 62.34 | ± 0.32 |
| Seranza | 41.49 | ± 0.23 | 48.39 | | ± 0.13 |  | 45.20 | ± 0.28 | 51.23 | ± 0.55 |
| Shamasbi | 59.51 | ± 0.03 | 68.21 | | ± 0.20 |  | 63.69 | ± 0.10 | 77.86 | ± 0.35 |
| TangeSehRiz1 | 68.06 | ± 0.03 | 75.83 | | ± 0.13 |  | 68.61 | ± 0.43 | 79.30 | ± 0.86 |
| Sileh | 51.86 | ± 0.06 | 65.78 | | ± 0.11 |  | 56.81 | ± 0.16 | 72.98 | ± 0.28 |
| SinavaCheshme | 33.83 | ± 0.22 | 42.40 | | ± 0.07 |  | 34.53 | ± 0.11 | 42.72 | ± 0.19 |
| Subashi | 47.38 | ± 0.15 | 54.47 | | ± 0.82 |  | 53.26 | ± 1.55 | 60.64 | ± 0.74 |
| Sureshjan | 53.73 | ± 0.20 | 65.35 | | ± 0.71 |  | 46.84 | ± 0.04 | 62.69 | ± 0.32 |
| Talesh | 31.70 | ± 0.06 | 41.67 | | ± 0.03 |  | 35.55 | ± 0.15 | 43.58 | ± 0.37 |
| TangeSehRiz2 | 58.34 | ± 0.60 | 76.54 | | ± 0.71 |  | 68.92 | ± 0.09 | 79.84 | ± 0.29 |
| TangeTizab | 43.84 | ± 0.06 | 49.76 | | ± 0.50 |  | 49.78 | ± 0.15 | 62.09 | ± 0.82 |
| Tangrah | 35.20 | ± 0.14 | 42.90 | | ± 0.13 |  | 40.39 | ± 0.04 | 44.21 | ± 0.21 |
| Tazehabad | 30.17 | ± 0.09 | 36.64 | | ± 0.06 |  | 33.96 | ± 0.02 | 42.27 | ± 0.01 |
| Telochal | 36.28 | ± 0.16 | 43.83 | | ± 0.29 |  | 44.23 | ± 0.51 | 53.01 | ± 0.78 |
| Tokhmaqlu | 44.38 | ± 0.00 | 49.16 | | ± 0.13 |  | 45.56 | ± 0.06 | 54.00 | ± 0.18 |
| Torshab | 68.84 | ± 0.02 | 74.26 | | ± 0.32 |  | 68.97 | ± 0.21 | 80.35 | ± 0.16 |
| Vanehbin | 56.40 | ± 0.03 | 62.85 | | ± 0.03 |  | 58.49 | ± 5.04 | 62.59 | ± 1.44 |
| Vila Darre Waterfall | 63.76 | ± 0.11 | 74.63 | | ± 0.13 |  | 71.25 | ± 0.34 | 83.84 | ± 0.42 |
| Yasuj | 52.24 | ± 1.31 | 58.52 | | ± 3.57 |  | 58.08 | ± 0.39 | 70.38 | ± 1.25 |
| Ziarat | 36.12 | ± 1.01 | 42.55 | | ± 1.32 |  | 45.53 | ± 0.05 | 54.02 | ± 0.77 |
| G | Irrigated | | | | |  | Non-irrigated | | | |
|  | 2018 | | 2019 | | |  | 2018 | | 2019 | |
|  | Antho | ± SE | Antho | | ± SE |  | Antho | ± SE | Antho | ± SE |
| Abbasabad | 17.03 | ± 0.37 | 24.13 | | ± 0.04 |  | 23.10 | ± 0.10 | 27.09 | ± 0.72 |
| Abr1Forest1 | 40.71 | ± 0.06 | 50.48 | | ± 0.21 |  | 48.57 | ± 0.17 | 54.15 | ± 0.93 |
| Abr2Forest2 | 37.27 | ± 0.08 | 42.14 | | ± 0.99 |  | 41.80 | ± 0.09 | 41.01 | ± 0.24 |
| Abr3Forest3 | 50.93 | ± 6.43 | 54.00 | | ± 8.82 |  | 50.48 | ± 0.05 | 63.89 | ± 0.01 |
| Abr1 | 34.47 | ± 0.07 | 42.26 | | ± 0.02 |  | 29.31 | ± 0.03 | 34.68 | ± 0.08 |
| Abr2 | 33.92 | ± 0.49 | 42.24 | | ± 0.89 |  | 36.84 | ± 0.03 | 49.65 | ± 0.34 |
| Abrumand | 34.20 | ± 0.01 | 51.83 | | ± 0.00 |  | 54.26 | ± 5.99 | 56.63 | ± 7.90 |
| Ahangaran | 36.74 | ± 0.13 | 42.07 | | ± 0.48 |  | 39.74 | ± 0.33 | 48.43 | ± 0.08 |
| Alvaresi | 34.76 | ± 0.26 | 41.22 | | ± 0.72 |  | 33.23 | ± 0.71 | 44.40 | ± 0.28 |
| Asadli | 32.91 | ± 0.06 | 38.46 | | ± 0.07 |  | 43.65 | ± 0.03 | 53.89 | ± 0.35 |
| Ashab | 35.99 | ± 0.51 | 47.42 | | ± 0.06 |  | 37.30 | ± 0.03 | 48.47 | ± 0.65 |
| Azizabad | 39.96 | ± 0.04 | 50.19 | | ± 0.14 |  | 42.23 | ± 0.17 | 51.61 | ± 0.03 |
| Aznãvleh | 25.82 | ± 0.61 | 32.58 | | ± 0.08 |  | 26.39 | ± 0.29 | 31.57 | ± 0.09 |
| BadKhoreh | 42.74 | ± 0.15 | 49.50 | | ± 0.02 |  | 27.81 | ± 0.00 | 52.98 | ± 0.10 |
| BandarehAnzali | 20.20 | ± 0.12 | 24.81 | | ± 0.04 |  | 28.18 | ± 0.02 | 30.54 | ± 0.06 |
| Baneh | 42.51 | ± 0.14 | 49.08 | | ± 0.06 |  | 46.56 | ± 0.08 | 53.75 | ± 0.15 |
| Basmenj | 43.35 | ± 0.02 | 58.37 | | ± 0.10 |  | 49.34 | ± 0.05 | 57.62 | ± 0.38 |
| Beyraq | 35.36 | ± 0.02 | 49.27 | | ± 0.27 |  | 30.22 | ± 0.82 | 37.22 | ± 0.19 |
| Bisotun | 38.71 | ± 0.04 | 37.74 | | ± 0.19 |  | 41.74 | ± 0.12 | 50.31 | ± 0.08 |
| Borbor | 39.25 | ± 0.50 | 44.48 | | ± 0.02 |  | 48.05 | ± 0.05 | 52.28 | ± 0.02 |
| Borhan | 41.73 | ± 0.36 | 49.29 | | ± 0.01 |  | 49.61 | ± 0.32 | 54.58 | ± 0.38 |
| Chadegan | 32.37 | ± 1.24 | 40.16 | | ± 0.51 |  | 38.43 | ± 0.85 | 48.66 | ± 0.11 |
| Chaleki | 21.46 | ± 0.07 | 28.18 | | ± 0.02 |  | 23.83 | ± 0.08 | 26.64 | ± 0.06 |
| Chali | 34.91 | ± 0.67 | 45.86 | | ± 0.22 |  | 39.56 | ± 0.04 | 50.01 | ± 0.28 |
| Chavarchin | 39.30 | ± 1.21 | 46.04 | | ± 0.01 |  | 41.19 | ± 0.23 | 50.16 | ± 0.07 |
| Ciakhor | 48.56 | ± 0.00 | 59.56 | | ± 0.02 |  | 57.82 | ± 0.05 | 67.82 | ± 0.05 |
| Damavand | 30.66 | ± 0.76 | 51.41 | | ± 0.42 |  | 49.77 | ± 0.00 | 58.13 | ± 0.05 |
| Damghanat | 30.59 | ± 0.13 | 35.27 | | ± 0.04 |  | 37.99 | ± 0.31 | 48.52 | ± 0.00 |
| Darband | 45.14 | ± 0.19 | 51.61 | | ± 0.59 |  | 44.20 | ± 0.04 | 52.58 | ± 0.07 |
| DareSari | 30.12 | ± 0.34 | 33.18 | | ± 0.21 |  | 33.70 | ± 0.06 | 40.44 | ± 0.71 |
| Darman | 44.61 | ± 0.08 | 50.83 | | ± 0.11 |  | 47.95 | ± 0.27 | 54.98 | ± 0.38 |
| DarrehLak | 38.74 | ± 0.16 | 50.69 | | ± 0.59 |  | 40.31 | ± 0.56 | 50.96 | ± 0.68 |
| DoSar | 31.51 | ± 0.09 | 52.60 | | ± 0.94 |  | 50.57 | ± 1.02 | 62.10 | ± 0.02 |
| Dowlatabad | 38.64 | ± 0.07 | 49.50 | | ± 0.12 |  | 36.42 | ± 0.19 | 45.77 | ± 0.24 |
| Duzduzan | 45.00 | ± 0.08 | 50.02 | | ± 0.25 |  | 45.50 | ± 0.08 | 52.10 | ± 0.07 |
| EisaKand | 40.49 | ± 0.16 | 46.05 | | ± 0.07 |  | 48.51 | ± 0.12 | 51.77 | ± 0.47 |
| Filabad | 33.40 | ± 0.38 | 44.74 | | ± 0.21 |  | 32.57 | ± 0.57 | 42.31 | ± 1.43 |
| Gaznaq | 45.76 | ± 0.12 | 51.77 | | ± 0.22 |  | 48.37 | ± 1.39 | 59.08 | ± 0.29 |
| Ghircanyon1 | 20.90 | ± 0.56 | 27.58 | | ± 0.01 |  | 24.13 | ± 0.38 | 34.37 | ± 0.04 |
| GilanTappeh | 21.92 | ± 0.59 | 28.75 | | ± 0.07 |  | 22.21 | ± 0.40 | 27.90 | ± 0.00 |
| Goorsephid | 31.41 | ± 0.12 | 34.24 | | ± 0.01 |  | 41.33 | ± 0.34 | 50.37 | ± 0.28 |
| Hamashahr | 39.97 | ± 0.06 | 42.44 | | ± 0.07 |  | 42.74 | ± 0.02 | 52.34 | ± 0.07 |
| Hamedan | 42.95 | ± 0.06 | 49.91 | | ± 0.01 |  | 49.48 | ± 0.07 | 55.39 | ± 0.15 |
| HasanQeshlaq | 42.28 | ± 0.12 | 48.78 | | ± 0.04 |  | 47.01 | ± 0.24 | 56.74 | ± 0.05 |
| Heyran | 31.70 | ± 0.1 | 35.71 | | ± 0.10 |  | 37.38 | ± 0.13 | 43.77 | ± 0.79 |
| Hezarkanian | 42.04 | ± 0.03 | 48.31 | | ± 0.09 |  | 48.55 | ± 0.64 | 53.66 | ± 0.14 |
| Isparaxan | 32.44 | ± 0.19 | 35.89 | | ± 0.30 |  | 33.24 | ± 0.04 | 37.08 | ± 0.24 |
| Kalat | 46.50 | ± 0.00 | 53.55 | | ± 2.00 |  | 39.21 | ± 0.14 | 49.46 | ± 0.23 |
| KalatehNaqi | 36.80 | ± 0.01 | 45.68 | | ± 0.23 |  | 43.08 | ± 0.31 | 49.20 | ± 0.19 |
| KaniGanji | 22.68 | ± 0.00 | 42.50 | | ± 0.01 |  | 43.33 | ± 0.18 | 52.10 | ± 0.01 |
| Kargan | 43.16 | ± 0.07 | 50.93 | | ± 1.14 |  | 42.92 | ± 0.06 | 51.70 | ± 0.07 |
| Karimabad | 18.05 | ± 0.02 | 24.09 | | ± 0.16 |  | 22.01 | ± 0.72 | 28.46 | ± 0.01 |
| Karkaraq | 39.89 | ± 0.16 | 47.10 | | ± 0.04 |  | 42.33 | ± 0.66 | 50.63 | ± 0.15 |
| Karvandan | 45.43 | ± 0.71 | 54.22 | | ± 0.61 |  | 51.71 | ± 0.10 | 68.29 | ± 1.26 |
| Kelardasht | 34.04 | ± 0.18 | 40.53 | | ± 0.20 |  | 40.20 | ± 0.52 | 48.10 | ± 1.38 |
| Khorramabad | 25.80 | ± 0.11 | 32.26 | | ± 0.03 |  | 30.03 | ± 0.09 | 34.76 | ± 0.03 |
| Khosrowabad | 40.55 | ± 0.10 | 45.61 | | ± 0.02 |  | 48.33 | ± 0.29 | 54.48 | ± 0.21 |
| KusehKahriz | 37.68 | ± 0.09 | 47.16 | | ± 0.48 |  | 39.21 | ± 0.22 | 46.05 | ± 0.73 |
| Laj | 33.36 | ± 0.07 | 35.67 | | ± 0.17 |  | 41.74 | ± 0.14 | 49.14 | ± 0.28 |
| Lamis | 41.82 | ± 0.09 | 47.59 | | ± 0.19 |  | 43.77 | ± 0.08 | 46.64 | ± 0.54 |
| LasemCheshmeh | 37.30 | ± 0.15 | 42.17 | | ± 0.44 |  | 48.75 | ± 0.31 | 50.62 | ± 0.43 |
| Liqvan | 29.53 | ± 0.04 | 33.47 | | ± 0.04 |  | 27.01 | ± 0.07 | 40.30 | ± 0.08 |
| Losku | 17.97 | ± 0.75 | 24.45 | | ± 0.12 |  | 22.13 | ± 0.19 | 27.17 | ± 0.10 |
| Mamlejeh | 36.13 | ± 0.23 | 42.80 | | ± 0.14 |  | 41.58 | ± 0.18 | 48.84 | ± 0.08 |
| Marian | 20.26 | ± 1.04 | 24.72 | | ± 0.92 |  | 23.14 | ± 0.06 | 26.15 | ± 0.01 |
| MazraeBeed | 35.54 | ± 0.03 | 45.60 | | ± 0.02 |  | 45.01 | ± 0.43 | 53.23 | ± 0.32 |
| MihamlehyeOlya | 33.64 | ± 1.43 | 46.77 | | ± 0.53 |  | 39.57 | ± 0.26 | 50.05 | ± 0.10 |
| MirAzizi | 42.20 | ± 0.26 | 44.47 | | ± 0.01 |  | 39.62 | ± 0.54 | 47.96 | ± 0.27 |
| Naharkhoran | 23.10 | ± 0.07 | 28.98 | | ± 0.12 |  | 28.03 | ± 0.98 | 33.96 | ± 0.40 |
| Nasirabad | 31.16 | ± 0.12 | 34.05 | | ± 0.23 |  | 31.83 | ± 0.12 | 37.11 | ± 0.38 |
| Noqan | 31.33 | ± 0.00 | 34.61 | | ± 0.01 |  | 33.29 | ± 0.29 | 36.95 | ± 0.25 |
| Nowgaran | 33.25 | ± 1.10 | 43.38 | | ± 0.14 |  | 41.52 | ± 0.61 | 49.07 | ± 0.58 |
| PahnehBar | 42.05 | ± 0.03 | 47.76 | | ± 0.04 |  | 49.37 | ± 0.17 | 55.13 | ± 0.08 |
| Palam | 25.70 | ± 0.11 | 30.27 | | ± 0.19 |  | 26.22 | ± 0.17 | 30.49 | ± 0.13 |
| Qorveh | 41.06 | ± 0.13 | 46.03 | | ± 0.12 |  | 42.66 | ± 0.07 | 51.23 | ± 0.37 |
| Qozivand | 35.03 | ± 0.17 | 38.88 | | ± 0.11 |  | 36.17 | ± 0.15 | 41.77 | ± 0.18 |
| QuriChay | 42.22 | ± 0.63 | 47.59 | | ± 0.19 |  | 46.35 | ± 0.38 | 51.72 | ± 0.12 |
| RezaqoliyeQeshlaq | 40.61 | ± 0.25 | 49.10 | | ± 0.10 |  | 52.13 | ± 1.63 | 59.04 | ± 0.05 |
| Roodafshan | 30.16 | ± 0.08 | 37.49 | | ± 0.77 |  | 37.71 | ± 0.24 | 49.57 | ± 0.10 |
| Sarab | 29.79 | ± 0.09 | 49.44 | | ± 0.26 |  | 47.66 | ± 0.01 | 54.24 | ± 0.17 |
| Sarbandan | 27.08 | ± 0.63 | 33.49 | | ± 0.09 |  | 34.70 | ± 0.07 | 42.33 | ± 0.22 |
| Seranza | 28.17 | ± 0.15 | 32.86 | | ± 0.09 |  | 27.64 | ± 0.15 | 34.79 | ± 0.38 |
| Shamasbi | 40.41 | ± 0.02 | 46.32 | | ± 0.14 |  | 43.25 | ± 0.07 | 52.87 | ± 0.24 |
| TangeSehRiz1 | 31.10 | ± 0.48 | 51.50 | | ± 0.09 |  | 46.59 | ± 0.29 | 53.85 | ± 0.59 |
| Sileh | 35.22 | ± 0.04 | 44.67 | | ± 0.08 |  | 38.58 | ± 0.11 | 49.55 | ± 0.19 |
| SinavaCheshme | 22.98 | ± 0.15 | 28.79 | | ± 0.05 |  | 23.45 | ± 0.08 | 29.01 | ± 0.13 |
| Subashi | 32.17 | ± 0.10 | 36.99 | | ± 0.56 |  | 38.29 | ± 0.46 | 45.76 | ± 1.16 |
| Sureshjan | 36.48 | ± 0.14 | 44.37 | | ± 0.48 |  | 31.81 | ± 0.02 | 42.57 | ± 0.22 |
| Talesh | 21.53 | ± 0.04 | 28.30 | | ± 0.02 |  | 24.14 | ± 0.1 | 29.59 | ± 0.25 |
| TangeSehRiz2 | 31.40 | ± 0.19 | 51.97 | | ± 0.48 |  | 46.80 | ± 0.06 | 54.22 | ± 0.20 |
| TangeTizab | 29.77 | ± 0.04 | 33.79 | | ± 0.34 |  | 33.80 | ± 0.1 | 42.16 | ± 0.56 |
| Tangrah | 25.29 | ± 0.02 | 31.52 | | ± 0.02 |  | 27.43 | ± 0.02 | 33.20 | ± 0.03 |
| Tazehabad | 20.48 | ± 0.06 | 24.88 | | ± 0.04 |  | 23.06 | ± 0.01 | 28.70 | ± 0.01 |
| Telochal | 24.63 | ± 0.11 | 29.76 | | ± 0.19 |  | 30.04 | ± 0.35 | 36.00 | ± 0.53 |
| Tokhmaqlu | 30.13 | ± 0.00 | 33.38 | | ± 0.09 |  | 30.94 | ± 0.04 | 36.67 | ± 0.13 |
| Torshab | 46.74 | ± 0.01 | 50.43 | | ± 0.22 |  | 46.83 | ± 0.14 | 54.56 | ± 0.11 |
| Vanehbin | 38.30 | ± 0.02 | 42.68 | | ± 0.02 |  | 40.32 | ± 1.03 | 53.52 | ± 0.04 |
| Vila Darre Waterfall | 43.29 | ± 0.07 | 50.68 | | ± 0.09 |  | 48.39 | ± 0.23 | 56.93 | ± 0.29 |
| Yasuj | 37.47 | ± 1.11 | 46.40 | | ± 2.54 |  | 39.44 | ± 0.27 | 47.79 | ± 0.85 |
| Ziarat | 24.53 | ± 0.69 | 28.89 | | ± 0.89 |  | 30.92 | ± 0.03 | 36.68 | ± 0.52 |
| G | Irrigated | | | | |  | Non-irrigated | | | |
|  | 2018 | | 2019 | | |  | 2018 | | 2019 | |
|  | FFY | ± SE | FFY | | ± SE |  | FFY | ± SE | FFY | ± SE |
| Abbasabad | 488.40 | ± 6.37 | 920.01 | ± 9.47 | |  | 346.00 | ± 6.00 | 672.91 | ± 5.99 |
| Abr1Forest1 | 645.50 | ± 14.66 | 1264.50 | ± 14.17 | |  | 351.56 | ± 7.40 | 796.08 | ± 3.02 |
| Abr2Forest2 | 653.95 | ± 0.95 | 1315.70 | ± 7.59 | |  | 500.69 | ± 4.31 | 1084.38 | ± 8.62 |
| Abr3Forest3 | 1488.04 | ± 515.63 | 1472.10 | ± 512.34 | |  | 611.07 | ± 4.34 | 1252.75 | ± 7.75 |
| Abr1 | 628.21 | ± 7.25 | 1243.82 | ± 42.02 | |  | 440.95 | ± 3.64 | 1024.61 | ± 17.44 |
| Abr2 | 631.93 | ± 0.27 | 1272.75 | ± 15.11 | |  | 465.89 | ± 5.15 | 989.82 | ± 2.85 |
| Abrumand | 950.58 | ± 6.53 | 1927.23 | ± 6.58 | |  | 911.53 | ± 383.97 | 958.06 | ± 370.88 |
| Ahangaran | 734.30 | ± 2.30 | 1490.86 | ± 16.71 | |  | 471.68 | ± 3.45 | 1015.68 | ± 0.26 |
| Alvaresi | 701.66 | ± 1.09 | 1898.72 | ± 2.46 | |  | 444.15 | ± 6.17 | 979.52 | ± 0.44 |
| Asadli | 733.65 | ± 2.45 | 1490.14 | ± 4.85 | |  | 594.10 | ± 1.10 | 1121.43 | ± 1.57 |
| Ashab | 449.46 | ± 7.5 | 1314.69 | ± 11.64 | |  | 422.50 | ± 7.55 | 1016.43 | ± 6.54 |
| Azizabad | 760.82 | ± 3.18 | 1528.29 | ± 13.70 | |  | 509.46 | ± 0.03 | 1089.21 | ± 2.96 |
| Aznãvleh | 630.42 | ± 3.93 | 1708.69 | ± 1.72 | |  | 423.20 | ± 2.48 | 1122.19 | ± 6.03 |
| BadKhoreh | 861.98 | ± 0.59 | 1663.56 | ± 2.96 | |  | 502.77 | ± 8.71 | 904.88 | ± 2.12 |
| BandarehAnzali | 518.59 | ± 1.71 | 1092.17 | ± 32.96 | |  | 396.88 | ± 18.15 | 880.06 | ± 7.96 |
| Baneh | 849.56 | ± 7.56 | 1315.76 | ± 10.26 | |  | 520.37 | ± 0.73 | 783.50 | ± 5.50 |
| Basmenj | 894.96 | ± 6.04 | 1886.19 | ± 4.01 | |  | 572.22 | ± 13.34 | 1229.92 | ± 4.08 |
| Beyraq | 656.11 | ± 4.64 | 1629.88 | ± 1.52 | |  | 435.31 | ± 0.49 | 941.11 | ± 0.52 |
| Bisotun | 732.11 | ± 6.07 | 1370.88 | ± 7.09 | |  | 494.66 | ± 8.13 | 1103.32 | ± 2.32 |
| Borbor | 730.16 | ± 8.36 | 1389.11 | ± 15.19 | |  | 503.81 | ± 5.84 | 1109.05 | ± 13.95 |
| Borhan | 882.64 | ± 2.36 | 1298.87 | ± 7.76 | |  | 532.47 | ± 0.18 | 1234.05 | ± 9.95 |
| Chadegan | 759.03 | ± 39.43 | 1418.74 | ± 1.29 | |  | 465.60 | ± 0.70 | 1016.67 | ± 3.56 |
| Chaleki | 495.70 | ± 6.35 | 1090.64 | ± 32.30 | |  | 406.53 | ± 3.23 | 837.20 | ± 8.54 |
| Chali | 738.47 | ± 0.59 | 1269.93 | ± 18.52 | |  | 486.00 | ± 10.00 | 1073.00 | ± 2.00 |
| Chavarchin | 735.43 | ± 4.14 | 1398.69 | ± 4.53 | |  | 512.49 | ± 5.51 | 1082.60 | ± 5.60 |
| Ciakhor | 969.40 | ± 1.91 | 2026.57 | ± 9.15 | |  | 627.66 | ± 10.02 | 1290.58 | ± 0.80 |
| Damavand | 890.67 | ± 4.33 | 1864.01 | ± 4.00 | |  | 646.01 | ± 3.65 | 1339.80 | ± 1.80 |
| Damghanat | 807.16 | ± 5.78 | 1792.07 | ± 0.40 | |  | 494.61 | ± 8.39 | 1055.70 | ± 2.10 |
| Darband | 624.67 | ± 7.33 | 1282.74 | ± 1.27 | |  | 529.98 | ± 11.37 | 970.75 | ± 2.85 |
| DareSari | 652.68 | ± 7.91 | 1852.43 | ± 12.39 | |  | 440.71 | ± 4.64 | 956.23 | ± 9.51 |
| Darman | 878.38 | ± 1.92 | 1712.59 | ± 4.56 | |  | 460.89 | ± 4.61 | 849.72 | ± 38.72 |
| DarrehLak | 708.28 | ± 4.71 | 1437.70 | ± 16.20 | |  | 595.46 | ± 6.20 | 1114.32 | ± 3.59 |
| DoSar | 882.27 | ± 1.74 | 1839.89 | ± 12.27 | |  | 610.97 | ± 3.39 | 1290.43 | ± 6.57 |
| Dowlatabad | 842.96 | ± 2.04 | 1347.84 | ± 1.52 | |  | 538.43 | ± 15.67 | 1186.10 | ± 4.90 |
| Duzduzan | 686.79 | ± 4.21 | 1392.33 | ± 5.81 | |  | 552.24 | ± 17.75 | 1177.53 | ± 13.53 |
| EisaKand | 772.29 | ± 12.78 | 1566.29 | ± 9.11 | |  | 525.58 | ± 10.99 | 1128.93 | ± 1.51 |
| Filabad | 633.79 | ± 0.31 | 1272.39 | ± 9.39 | |  | 456.22 | ± 0.37 | 1013.97 | ± 6.10 |
| Gaznaq | 682.05 | ± 13.95 | 1358.42 | ± 16.2 | |  | 539.00 | ± 8.00 | 1178.94 | ± 6.94 |
| Ghircanyon1 | 496.73 | ± 29.07 | 1102.61 | ± 1.65 | |  | 431.52 | ± 0.58 | 933.59 | ± 8.31 |
| GilanTappeh | 523.29 | ± 5.64 | 1301.91 | ± 6.54 | |  | 332.50 | ± 2.50 | 646.00 | ± 4.00 |
| Goorsephid | 634.77 | ± 13.06 | 1851.06 | ± 37.34 | |  | 431.30 | ± 0.70 | 1057.44 | ± 4.56 |
| Hamashahr | 788.40 | ± 9.65 | 1501.70 | ± 3.84 | |  | 519.07 | ± 6.93 | 1129.21 | ± 13.79 |
| Hamedan | 677.94 | ± 0.71 | 1793.80 | ± 0.29 | |  | 615.69 | ± 9.71 | 1253.69 | ± 1.46 |
| HasanQeshlaq | 853.23 | ± 2.77 | 1239.47 | ± 1.82 | |  | 587.08 | ± 12.93 | 1211.04 | ± 2.04 |
| Heyran | 588.14 | ± 4.68 | 1729.17 | ± 1.49 | |  | 430.08 | ± 2.07 | 934.82 | ± 8.16 |
| Hezarkanian | 910.69 | ± 3.31 | 1243.79 | ± 4.56 | |  | 586.07 | ± 18.17 | 1220.45 | ± 1.52 |
| Isparaxan | 602.06 | ± 3.60 | 1829.84 | ± 12.6 | |  | 538.24 | ± 2.24 | 1008.60 | ± 4.40 |
| Kalat | 740.56 | ± 0.40 | 1381.70 | ± 2.34 | |  | 495.00 | ± 3.00 | 1061.83 | ± 3.83 |
| KalatehNaqi | 692.16 | ± 7.90 | 1376.12 | ± 14.43 | |  | 488.01 | ± 0.55 | 1067.71 | ± 9.29 |
| KaniGanji | 628.36 | ± 5.45 | 1390.08 | ± 4.56 | |  | 528.78 | ± 12.38 | 1138.73 | ± 2.79 |
| Kargan | 805.90 | ± 6.08 | 1568.27 | ± 5.35 | |  | 526.78 | ± 12.91 | 1114.41 | ± 4.41 |
| Karimabad | 552.80 | ± 11.10 | 1117.49 | ± 12.07 | |  | 378.46 | ± 2.54 | 866.54 | ± 10.11 |
| Karkaraq | 746.81 | ± 0.00 | 1466.81 | ± 5.82 | |  | 485.95 | ± 6.95 | 1080.00 | ± 3.00 |
| Karvandan | 889.86 | ± 4.86 | 1904.96 | ± 10.63 | |  | 613.62 | ± 8.44 | 1316.61 | ± 1.39 |
| Kelardasht | 731.74 | ± 4.41 | 1425.71 | ± 10.28 | |  | 457.66 | ± 17.65 | 1048.29 | ± 4.34 |
| Khorramabad | 580.44 | ± 22.65 | 1644.32 | ± 11.92 | |  | 440.02 | ± 1.13 | 1009.73 | ± 4.64 |
| Khosrowabad | 770.12 | ± 23.84 | 1491.61 | ± 2.52 | |  | 503.53 | ± 8.47 | 1080.57 | ± 7.43 |
| KusehKahriz | 817.55 | ± 13.03 | 1239.06 | ± 6.25 | |  | 506.81 | ± 13.19 | 1070.72 | ± 0.26 |
| Laj | 729.64 | ± 6.60 | 1434.48 | ± 6.55 | |  | 451.05 | ± 10.49 | 1025.91 | ± 14.82 |
| Lamis | 846.40 | ± 9.60 | 1593.12 | ± 1.52 | |  | 489.25 | ± 1.25 | 1067.83 | ± 4.17 |
| LasemCheshmeh | 890.81 | ± 8.91 | 1744.56 | ± 9.11 | |  | 525.77 | ± 15.75 | 1116.63 | ± 8.37 |
| Liqvan | 580.17 | ± 25.89 | 1960.25 | ± 5.02 | |  | 441.28 | ± 7.04 | 940.79 | ± 10.69 |
| Losku | 495.40 | ± 3.00 | 968.10 | ± 15.30 | |  | 404.00 | ± 6.00 | 697.72 | ± 23.88 |
| Mamlejeh | 732.38 | ± 7.55 | 1381.23 | ± 6.86 | |  | 484.75 | ± 4.75 | 1058.57 | ± 3.57 |
| Marian | 539.27 | ± 14.86 | 1406.06 | ± 9.09 | |  | 409.62 | ± 6.99 | 915.83 | ± 2.31 |
| MazraeBeed | 480.00 | ± 5.96 | 990.09 | ± 6.24 | |  | 449.96 | ± 4.00 | 1053.50 | ± 2.50 |
| MihamlehyeOlya | 632.51 | ± 3.24 | 1893.93 | ± 2.02 | |  | 489.00 | ± 8.00 | 957.97 | ± 5.03 |
| MirAzizi | 728.78 | ± 4.76 | 1330.11 | ± 1.29 | |  | 479.02 | ± 0.49 | 1051.12 | ± 6.88 |
| Naharkhoran | 695.87 | ± 9.51 | 1802.50 | ± 5.50 | |  | 567.82 | ± 7.58 | 1095.74 | ± 3.98 |
| Nasirabad | 689.34 | ± 7.45 | 1879.98 | ± 0.69 | |  | 427.86 | ± 4.21 | 1105.84 | ± 1.36 |
| Noqan | 693.39 | ± 3.81 | 1906.60 | ± 9.73 | |  | 428.10 | ± 1.19 | 921.12 | ± 0.75 |
| Nowgaran | 562.04 | ± 3.96 | 1014.56 | ± 5.44 | |  | 453.55 | ± 13.66 | 997.68 | ± 2.32 |
| PahnehBar | 854.44 | ± 6.56 | 1609.32 | ± 5.57 | |  | 508.79 | ± 8.79 | 753.12 | ± 8.30 |
| Palam | 600.16 | ± 3.00 | 1697.91 | ± 1.01 | |  | 534.83 | ± 14.34 | 1093.57 | ± 2.02 |
| Qorveh | 804.37 | ± 7.94 | 1550.82 | ± 1.59 | |  | 528.26 | ± 14.14 | 1126.97 | ± 6.03 |
| Qozivand | 627.62 | ± 4.25 | 1422.52 | ± 15.19 | |  | 479.95 | ± 4.59 | 1049.92 | ± 3.65 |
| QuriChay | 843.82 | ± 6.52 | 1598.06 | ± 2.41 | |  | 547.09 | ± 19.99 | 711.10 | ± 2.08 |
| RezaqoliyeQeshlaq | 757.42 | ± 8.58 | 1479.22 | ± 2.02 | |  | 518.53 | ± 18.25 | 1094.65 | ± 9.35 |
| Roodafshan | 612.04 | ± 5.53 | 1207.74 | ± 7.97 | |  | 475.22 | ± 6.83 | 1018.45 | ± 2.08 |
| Sarab | 872.62 | ± 0.19 | 1694.95 | ± 1.95 | |  | 532.84 | ± 6.44 | 907.50 | ± 6.50 |
| Sarbandan | 587.33 | ± 25.6 | 1580.78 | ± 1.26 | |  | 424.00 | ± 4.00 | 916.33 | ± 1.23 |
| Seranza | 544.13 | ± 3.87 | 1611.58 | ± 11.37 | |  | 432.00 | ± 6.67 | 913.61 | ± 0.31 |
| Shamasbi | 749.44 | ± 4.56 | 1448.17 | ± 3.38 | |  | 490.59 | ± 6.71 | 1061.61 | ± 17.61 |
| TangeSehRiz1 | 599.24 | ± 14.24 | 1235.94 | ± 8.67 | |  | 537.38 | ± 6.63 | 1202.12 | ± 1.71 |
| Sileh | 631.96 | ± 4.69 | 1311.81 | ± 12.81 | |  | 453.07 | ± 13.67 | 1028.06 | ± 14.77 |
| SinavaCheshme | 489.58 | ± 13.90 | 1128.50 | ± 2.50 | |  | 406.91 | ± 6.65 | 986.93 | ± 10.25 |
| Subashi | 693.87 | ± 1.74 | 1842.27 | ± 3.24 | |  | 439.85 | ± 6.60 | 938.10 | ± 11.44 |
| Sureshjan | 624.94 | ± 4.68 | 1713.19 | ± 13.93 | |  | 452.44 | ± 2.31 | 943.95 | ± 18.34 |
| Talesh | 505.96 | ± 4.56 | 1053.50 | ± 4.50 | |  | 357.50 | ± 6.50 | 827.44 | ± 0.21 |
| TangeSehRiz2 | 606.02 | ± 23.83 | 1287.91 | ± 2.91 | |  | 546.86 | ± 22.76 | 872.55 | ± 11.55 |
| TangeTizab | 615.84 | ± 4.16 | 1659.28 | ± 13.16 | |  | 445.01 | ± 10.64 | 934.31 | ± 3.49 |
| Tangrah | 762.37 | ± 17.42 | 1785.54 | ± 0.46 | |  | 487.93 | ± 11.51 | 1002.77 | ± 1.29 |
| Tazehabad | 514.09 | ± 1.82 | 1082.13 | ± 6.57 | |  | 362.54 | ± 4.46 | 669.02 | ± 2.99 |
| Telochal | 586.80 | ± 7.69 | 1785.46 | ± 7.54 | |  | 433.47 | ± 8.73 | 906.81 | ± 2.35 |
| Tokhmaqlu | 541.84 | ± 18.31 | 1801.53 | ± 4.60 | |  | 532.58 | ± 9.67 | 1088.68 | ± 1.59 |
| Torshab | 891.56 | ± 5.44 | 1774.83 | ± 12.18 | |  | 564.90 | ± 7.97 | 1191.26 | ± 1.74 |
| Vanehbin | 728.42 | ± 8.47 | 1569.83 | ± 14.68 | |  | 476.45 | ± 1.01 | 1050.82 | ± 6.65 |
| Vila Darre Waterfall | 689.36 | ± 4.64 | 1474.98 | ± 5.18 | |  | 579.34 | ± 2.63 | 1235.67 | ± 0.75 |
| Yasuj | 864.43 | ± 4.58 | 1121.95 | ± 5.39 | |  | 453.41 | ± 14.51 | 1026.65 | ± 12.28 |
| Ziarat | 677.15 | ± 5.89 | 1804.90 | ± 11.01 | |  | 418.48 | ± 6.07 | 890.23 | ± 7.29 |
| G | Irrigated | | | | |  | Non-irrigated | | | |
|  | 2018 | | 2019 | | |  | 2018 | | 2019 | |
|  | FDY | ± SE | FDY | | ± SE |  | FDY | ± SE | FDY | ± SE |
| Abbasabad | 234.00 | ± 1.00 | 456.00 | | ± 2.00 |  | 201.94 | ± 3.18 | 380.88 | ± 9.38 |
| Abr1Forest1 | 351.86 | ± 5.93 | 693.50 | | ± 3.50 |  | 258.33 | ± 2.18 | 431.58 | ± 4.16 |
| Abr2Forest2 | 391.88 | ± 3.44 | 769.00 | | ± 1.00 |  | 263.02 | ± 3.20 | 687.70 | ± 15.25 |
| Abr3Forest3 | 783.28 | ± 31.37 | 820.38 | | ± 31.35 |  | 381.90 | ± 0.50 | 775.50 | ± 3.95 |
| Abr1 | 318.45 | ± 4.89 | 733.00 | | ± 13.00 |  | 237.76 | ± 2.96 | 598.64 | ± 13.72 |
| Abr2 | 360.05 | ± 1.81 | 743.00 | | ± 4.00 |  | 309.57 | ± 4.15 | 568.30 | ± 1.47 |
| Abrumand | 611.44 | ± 2.83 | 1082.50 | | ± 1.50 |  | 518.83 | ± 269.65 | 602.49 | ± 267.33 |
| Ahangaran | 335.72 | ± 5.70 | 841.00 | | ± 13.00 |  | 289.77 | ± 4.33 | 630.54 | ± 9.10 |
| Alvaresi | 372.56 | ± 10.56 | 1086.50 | | ± 29.50 |  | 241.70 | ± 14.46 | 566.16 | ± 2.39 |
| Asadli | 343.43 | ± 4.81 | 843.50 | | ± 3.50 |  | 334.96 | ± 2.21 | 680.09 | ± 0.16 |
| Ashab | 258.93 | ± 7.00 | 762.50 | | ± 4.50 |  | 196.00 | ± 4.79 | 595.39 | ± 5.95 |
| Azizabad | 345.45 | ± 8.55 | 860.50 | | ± 2.50 |  | 283.49 | ± 0.33 | 693.96 | ± 8.78 |
| Aznãvleh | 313.62 | ± 9.03 | 724.00 | | ± 2.00 |  | 222.22 | ± 0.45 | 667.14 | ± 11.77 |
| BadKhoreh | 347.39 | ± 4.39 | 966.50 | | ± 0.50 |  | 283.85 | ± 13.8 | 649.90 | ± 4.52 |
| BandarehAnzali | 256.57 | ± 4.71 | 522.50 | | ± 7.50 |  | 225.13 | ± 10.72 | 488.64 | ± 7.55 |
| Baneh | 347.61 | ± 8.39 | 705.00 | | ± 2.00 |  | 268.10 | ± 4.74 | 670.35 | ± 1.27 |
| Basmenj | 529.89 | ± 2.00 | 1043.50 | | ± 1.50 |  | 332.74 | ± 0.40 | 764.12 | ± 3.32 |
| Beyraq | 363.50 | ± 9.50 | 768.00 | | ± 6.00 |  | 236.93 | ± 5.01 | 530.73 | ± 0.12 |
| Bisotun | 335.13 | ± 7.83 | 781.50 | | ± 3.50 |  | 281.27 | ± 11.23 | 703.73 | ± 0.41 |
| Borbor | 334.61 | ± 8.64 | 788.50 | | ± 6.50 |  | 282.68 | ± 0.85 | 711.68 | ± 5.89 |
| Borhan | 377.05 | ± 6.95 | 782.00 | | ± 1.00 |  | 315.85 | ± 4.57 | 775.37 | ± 7.66 |
| Chadegan | 327.92 | ± 6.30 | 792.50 | | ± 0.50 |  | 274.50 | ± 2.78 | 597.29 | ± 6.42 |
| Chaleki | 259.64 | ± 1.45 | 532.50 | | ± 19.50 |  | 213.60 | ± 1.22 | 449.56 | ± 7.38 |
| Chali | 372.39 | ± 3.03 | 744.50 | | ± 5.50 |  | 259.38 | ± 5.74 | 668.71 | ± 2.79 |
| Chavarchin | 338.56 | ± 6.8 | 788.50 | | ± 4.50 |  | 261.93 | ± 0.21 | 694.62 | ± 1.84 |
| Ciakhor | 541.28 | ± 8.28 | 1129.00 | | ± 3.00 |  | 375.84 | ± 7.35 | 801.67 | ± 3.45 |
| Damavand | 486.16 | ± 1.97 | 1029.50 | | ± 6.50 |  | 461.94 | ± 8.46 | 864.95 | ± 4.00 |
| Damghanat | 295.89 | ± 5.47 | 914.50 | | ± 3.50 |  | 254.08 | ± 4.48 | 667.49 | ± 1.01 |
| Darband | 325.58 | ± 8.58 | 737.50 | | ± 6.50 |  | 310.48 | ± 7.49 | 611.80 | ± 4.10 |
| DareSari | 351.78 | ± 3.42 | 1011.00 | | ± 17.00 |  | 232.25 | ± 2.31 | 539.70 | ± 1.34 |
| Darman | 354.86 | ± 5.71 | 815.00 | | ± 6.00 |  | 272.70 | ± 6.28 | 564.89 | ± 8.99 |
| DarrehLak | 486.22 | ± 1.78 | 814.50 | | ± 9.50 |  | 389.10 | ± 1.90 | 563.87 | ± 2.20 |
| DoSar | 417.31 | ± 15.37 | 1025.50 | | ± 5.50 |  | 281.37 | ± 2.59 | 830.22 | ± 0.38 |
| Dowlatabad | 398.15 | ± 6.15 | 747.00 | | ± 4.00 |  | 311.75 | ± 2.19 | 709.14 | ± 0.95 |
| Duzduzan | 367.12 | ± 0.88 | 816.50 | | ± 2.50 |  | 337.79 | ± 5.00 | 728.43 | ± 6.37 |
| EisaKand | 344.75 | ± 1.33 | 916.00 | | ± 10.00 |  | 318.44 | ± 10.96 | 660.85 | ± 0.89 |
| Filabad | 310.68 | ± 2.42 | 743.50 | | ± 0.50 |  | 259.53 | ± 2.49 | 599.08 | ± 7.10 |
| Gaznaq | 357.91 | ± 4.09 | 764.50 | | ± 2.50 |  | 319.56 | ± 9.70 | 635.85 | ± 14.64 |
| Ghircanyon1 | 250.14 | ± 9.55 | 547.50 | | ± 0.50 |  | 230.27 | ± 3.43 | 545.26 | ± 24.53 |
| GilanTappeh | 248.27 | ± 1.32 | 569.50 | | ± 9.50 |  | 175.54 | ± 6.91 | 357.48 | ± 8.77 |
| Goorsephid | 351.50 | ± 2.68 | 974.00 | | ± 22.00 |  | 239.35 | ± 1.46 | 661.34 | ± 3.13 |
| Hamashahr | 347.07 | ± 1.73 | 855.00 | | ± 5.00 |  | 309.66 | ± 0.97 | 673.61 | ± 7.88 |
| Hamedan | 365.66 | ± 4.47 | 828.00 | | ± 15.00 |  | 352.31 | ± 0.57 | 804.88 | ± 17.77 |
| HasanQeshlaq | 430.30 | ± 8.30 | 772.91 | | ± 0.50 |  | 314.86 | ± 12.23 | 558.50 | ± 0.36 |
| Heyran | 320.30 | ± 12.69 | 917.50 | | ± 14.50 |  | 239.53 | ± 10.85 | 546.73 | ± 13.21 |
| Hezarkanian | 448.40 | ± 6.60 | 776.11 | | ± 15.50 |  | 322.04 | ± 4.33 | 747.50 | ± 3.26 |
| Isparaxan | 323.47 | ± 1.06 | 988.00 | | ± 10.00 |  | 290.68 | ± 7.72 | 583.40 | ± 5.08 |
| Kalat | 343.83 | ± 3.83 | 791.50 | | ± 4.50 |  | 259.56 | ± 4.59 | 675.39 | ± 1.01 |
| KalatehNaqi | 321.57 | ± 1.54 | 784.50 | | ± 7.50 |  | 286.20 | ± 1.54 | 668.94 | ± 10.24 |
| KaniGanji | 326.99 | ± 6.04 | 844.00 | | ± 6.00 |  | 302.00 | ± 0.68 | 685.85 | ± 6.53 |
| Kargan | 351.60 | ± 0.23 | 934.50 | | ± 9.50 |  | 305.87 | ± 5.64 | 696.55 | ± 8.24 |
| Karimabad | 264.50 | ± 5.50 | 493.50 | | ± 7.50 |  | 200.34 | ± 1.3 | 445.92 | ± 3.97 |
| Karkaraq | 331.32 | ± 0.18 | 830.50 | | ± 4.50 |  | 266.39 | ± 0.49 | 680.01 | ± 6.33 |
| Karvandan | 505.58 | ± 2.62 | 1076.00 | | ± 5.00 |  | 381.24 | ± 2.97 | 820.46 | ± 4.20 |
| Kelardasht | 333.99 | ± 6.19 | 808.50 | | ± 4.50 |  | 269.04 | ± 8.07 | 645.10 | ± 7.60 |
| Khorramabad | 292.97 | ± 3.36 | 906.00 | | ± 2.00 |  | 257.10 | ± 5.00 | 587.46 | ± 5.84 |
| Khosrowabad | 351.75 | ± 1.25 | 842.00 | | ± 1.00 |  | 261.18 | ± 5.38 | 690.53 | ± 2.24 |
| KusehKahriz | 421.24 | ± 0.34 | 683.85 | | ± 8.00 |  | 289.96 | ± 18.23 | 570.00 | ± 0.90 |
| Laj | 333.56 | ± 7.55 | 810.00 | | ± 4.00 |  | 238.18 | ± 0.37 | 600.15 | ± 21.92 |
| Lamis | 438.50 | ± 3.50 | 944.50 | | ± 4.50 |  | 257.98 | ± 0.5 | 630.71 | ± 7.45 |
| LasemCheshmeh | 415.87 | ± 7.05 | 976.50 | | ± 0.50 |  | 291.15 | ± 7.94 | 669.90 | ± 15.25 |
| Liqvan | 292.12 | ± 4.42 | 1023.00 | | ± 16.00 |  | 237.56 | ± 5.84 | 565.99 | ± 5.63 |
| Losku | 260.73 | ± 2.44 | 470.00 | | ± 2.00 |  | 195.66 | ± 4.11 | 422.83 | ± 28.13 |
| Mamlejeh | 336.39 | ± 9.81 | 785.00 | | ± 2.00 |  | 256.82 | ± 3.11 | 668.79 | ± 0.03 |
| Marian | 278.55 | ± 3.08 | 622.00 | | ± 7.00 |  | 222.18 | ± 4.91 | 462.77 | ± 3.58 |
| MazraeBeed | 341.38 | ± 2.45 | 663.36 | | ± 11.13 |  | 209.55 | ± 10.12 | 559.88 | ± 0.61 |
| MihamlehyeOlya | 365.34 | ± 1.76 | 953.00 | | ± 1.00 |  | 258.87 | ± 2.17 | 656.68 | ± 12.04 |
| MirAzizi | 330.46 | ± 4.23 | 769.50 | | ± 5.50 |  | 300.76 | ± 6.33 | 652.57 | ± 5.81 |
| Naharkhoran | 358.71 | ± 8.78 | 662.26 | | ± 5.50 |  | 351.12 | ± 40.33 | 625.50 | ± 1.85 |
| Nasirabad | 367.58 | ± 7.54 | 1002.00 | | ± 9.00 |  | 223.05 | ± 2.96 | 628.01 | ± 20.68 |
| Noqan | 365.14 | ± 0.97 | 995.50 | | ± 5.50 |  | 224.88 | ± 1.56 | 536.50 | ± 7.40 |
| Nowgaran | 280.08 | ± 2.92 | 577.71 | | ± 9.00 |  | 258.64 | ± 0.96 | 461.00 | ± 1.44 |
| PahnehBar | 443.12 | ± 6.88 | 958.00 | | ± 2.00 |  | 274.63 | ± 1.54 | 494.26 | ± 4.00 |
| Palam | 316.85 | ± 6.76 | 786.00 | | ± 2.00 |  | 315.50 | ± 11.39 | 679.99 | ± 2.54 |
| Qorveh | 350.28 | ± 3.10 | 904.00 | | ± 0.00 |  | 313.55 | ± 5.36 | 652.86 | ± 8.62 |
| Qozivand | 350.12 | ± 5.80 | 853.00 | | ± 51.00 |  | 286.94 | ± 1.12 | 658.65 | ± 11.03 |
| QuriChay | 443.00 | ± 4.00 | 952.50 | | ± 2.50 |  | 308.82 | ± 5.02 | 430.41 | ± 1.27 |
| RezaqoliyeQeshlaq | 340.00 | ± 6.00 | 826.50 | | ± 3.50 |  | 269.36 | ± 2.68 | 697.64 | ± 9.30 |
| Roodafshan | 299.84 | ± 1.70 | 719.50 | | ± 0.50 |  | 244.38 | ± 2.31 | 640.59 | ± 3.48 |
| Sarab | 348.85 | ± 8.85 | 769.00 | | ± 0.00 |  | 304.36 | ± 4.34 | 540.22 | ± 16.51 |
| Sarbandan | 295.84 | ± 5.18 | 715.00 | | ± 10.00 |  | 229.12 | ± 1.73 | 535.57 | ± 16.95 |
| Seranza | 280.25 | ± 4.10 | 831.50 | | ± 9.50 |  | 227.74 | ± 3.70 | 534.16 | ± 18.32 |
| Shamasbi | 355.24 | ± 4.76 | 819.50 | | ± 1.50 |  | 263.54 | ± 7.70 | 677.45 | ± 2.06 |
| TangeSehRiz1 | 327.63 | ± 7.63 | 758.50 | | ± 2.50 |  | 315.02 | ± 0.82 | 721.01 | ± 11.06 |
| Sileh | 309.78 | ± 4.86 | 755.50 | | ± 9.50 |  | 268.00 | ± 10.76 | 606.14 | ± 15.16 |
| SinavaCheshme | 258.11 | ± 3.22 | 560.00 | | ± 4.00 |  | 205.36 | ± 4.36 | 448.65 | ± 4.55 |
| Subashi | 384.78 | ± 6.79 | 889.50 | | ± 15.50 |  | 246.66 | ± 1.73 | 530.14 | ± 9.37 |
| Sureshjan | 348.59 | ± 3.03 | 833.00 | | ± 4.00 |  | 250.73 | ± 7.10 | 538.03 | ± 17.02 |
| Talesh | 262.47 | ± 1.66 | 505.50 | | ± 4.50 |  | 194.56 | ± 3.87 | 492.68 | ± 12.6 |
| TangeSehRiz2 | 354.19 | ± 4.50 | 681.50 | | ± 6.50 |  | 321.79 | ± 7.35 | 483.53 | ± 11.69 |
| TangeTizab | 293.90 | ± 4.90 | 908.50 | | ± 3.50 |  | 238.67 | ± 0.29 | 528.30 | ± 2.46 |
| Tangrah | 381.22 | ± 6.22 | 885.50 | | ± 8.50 |  | 297.42 | ± 2.32 | 584.65 | ± 21.92 |
| Tazehabad | 246.25 | ± 3.90 | 526.00 | | ± 1.00 |  | 180.78 | ± 3.20 | 358.71 | ± 2.69 |
| Telochal | 304.04 | ± 4.00 | 893.50 | | ± 3.50 |  | 229.04 | ± 1.73 | 553.46 | ± 0.82 |
| Tokhmaqlu | 289.97 | ± 1.75 | 940.00 | | ± 9.00 |  | 231.06 | ± 4.97 | 643.62 | ± 11.67 |
| Torshab | 416.11 | ± 4.11 | 1005.50 | | ± 7.50 |  | 315.47 | ± 7.47 | 737.31 | ± 1.32 |
| Vanehbin | 333.68 | ± 8.07 | 919.50 | | ± 14.50 |  | 286.67 | ± 0.29 | 653.98 | ± 8.26 |
| Vila Darre Waterfall | 357.33 | ± 7.67 | 858.50 | | ± 8.50 |  | 336.68 | ± 2.73 | 750.71 | ± 4.74 |
| Yasuj | 382.83 | ± 4.63 | 607.78 | | ± 1.50 |  | 261.66 | ± 10.16 | 470.50 | ± 9.29 |
| Ziarat | 347.64 | ± 4.62 | 998.00 | | ± 12.00 |  | 218.97 | ± 2.76 | 542.42 | ± 1.92 |
| TPC, total phenol content; TFC, total flavonoid content; FLv, flavanone; Anti, antioxidant activity; Antho, anthocyanin; FFY, forage fresh yield; FDY, forage dry weight; SE, standard error of the mean. | | | | | | | | | | |
